# Supplementary material for: A method for tissue-mask supported whole-body image registration in the UK Biobank
Source: Sci Rep. 2026 Jun 22;16:19383. doi: 10.1038/s41598-026-58409-x (PMC13287688; doi:10.1038/s41598-026-58409-x)
Supplement: Supplementary file 1 — Supplementary Material 1 [file 41598_2026_58409_MOESM1_ESM.docx]

A method for tissue-mask-supported whole-body
image registration in the UK Biobank

Yasemin Utkueri^a*^, Elin Lundström^a^, Håkan Ahlström^a,c^, Johan Öfverstedt^a^ & Joel Kullberg^a,b,c^

a Department of Surgical Sciences, Uppsala University, Sweden.

b Department of Surgical Sciences, SciLifeLab, Uppsala University, Sweden.

c Antaros Medical, Mölndal, Sweden

## Supplementary Material

Supplementary Figure A: Ablation study results for the optimal regularization weight.


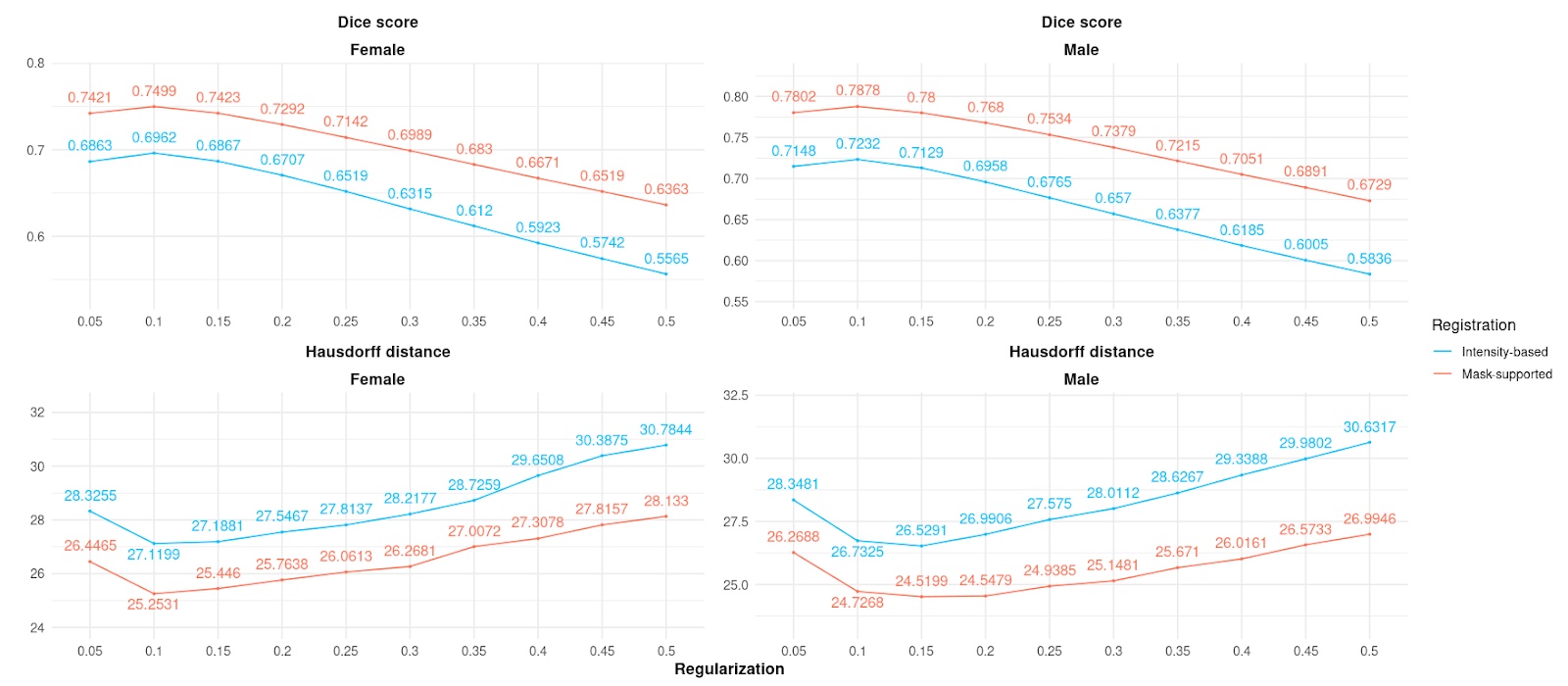


Mean dice scores and Hausdorff distances across the 71 masks and all subjects for different regularization weights for females and males. For the mask-supported registrations, the weight of the mask channel was set to 0.6 for these experiments.

Supplementary Figure B: Ablation study results to obtain the optimal weight for the SAT and muscle weights.


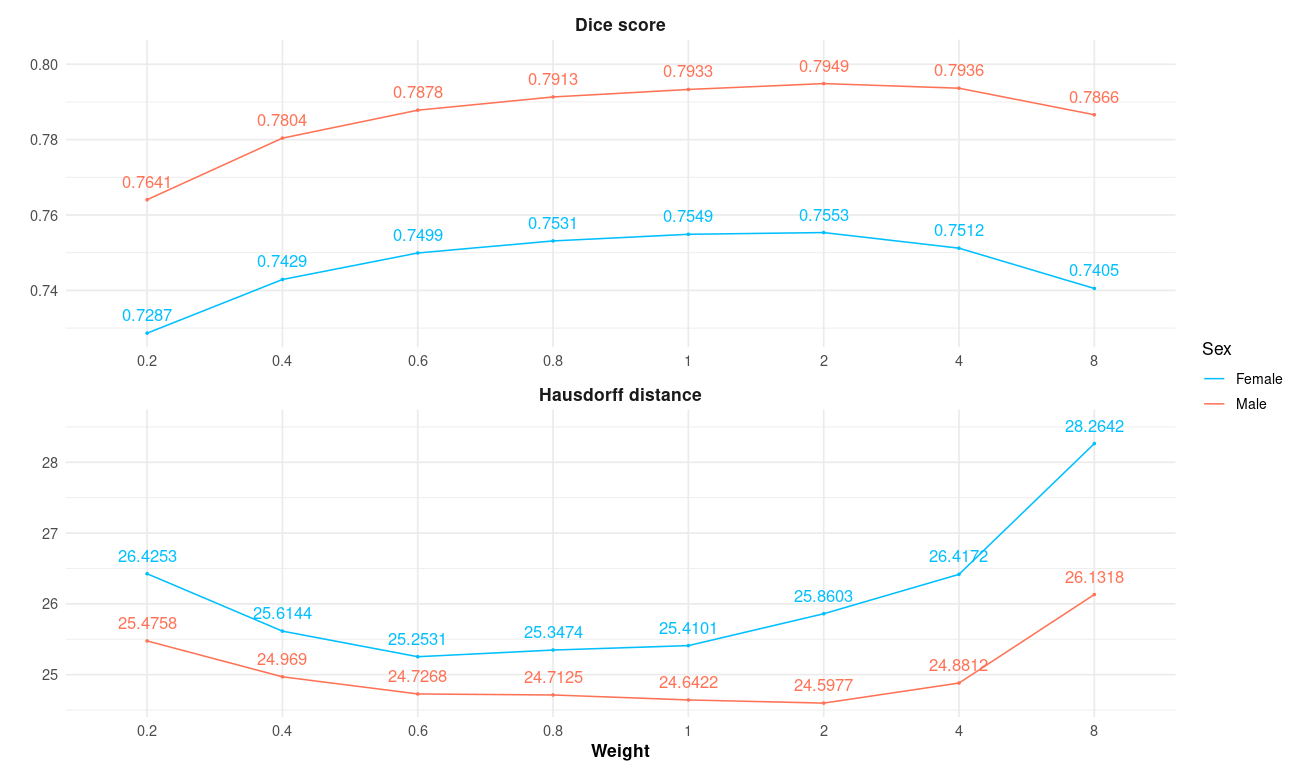


Mean dice scores and Hausdorff distances across the 71 masks and all subjects for different binary mask input channel weights for the mask-supported registration. The regularization weight for these experiments was set to 0.1.

Supplementary Figure C: Mean FF images for males and females registered with different mask channel weights


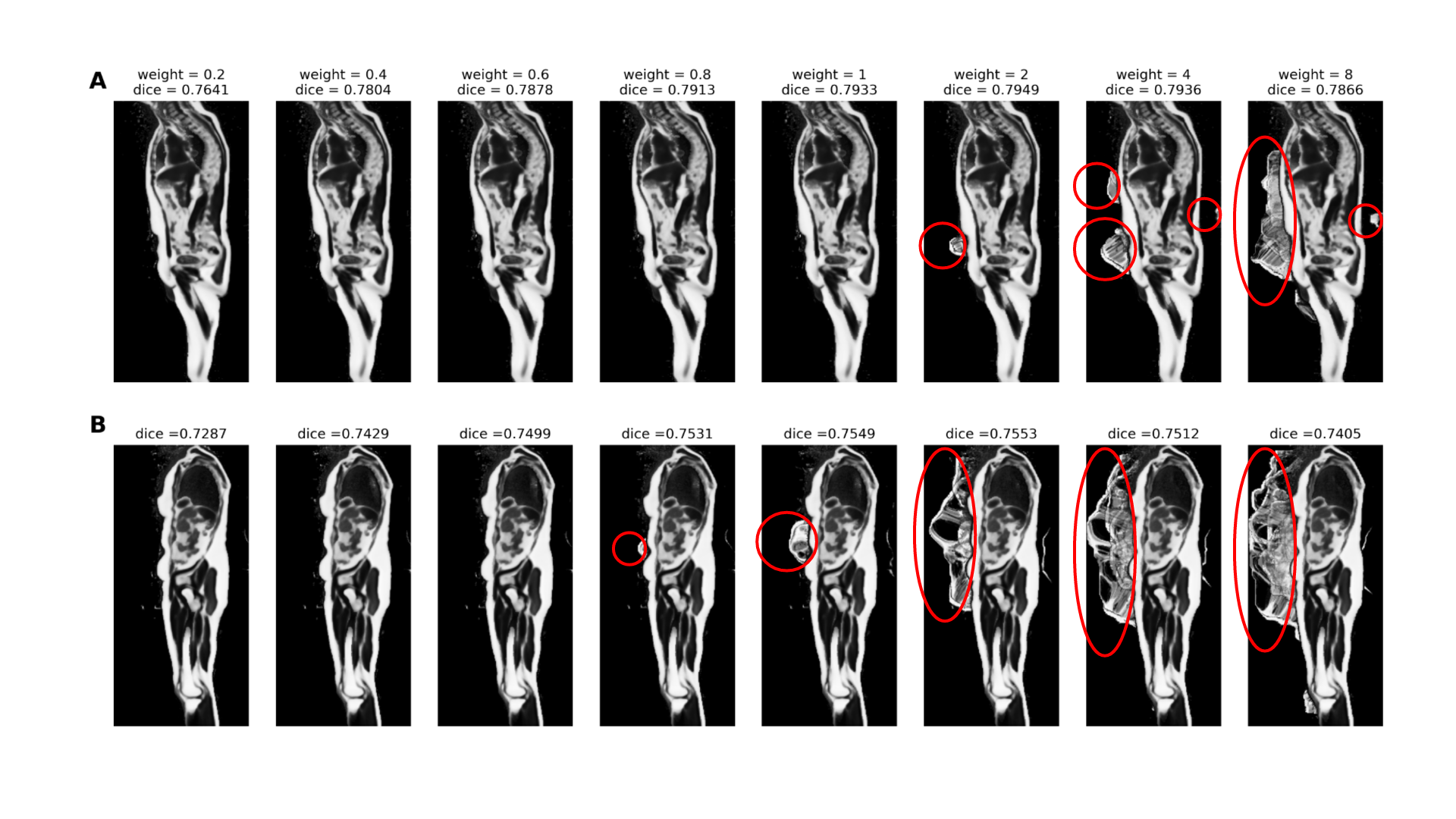


Mean FF images for the 200 males and females of the ablation sub-cohort that were registered using different mask channel weights. All registrations used 0.1 regularization. The observed artifacts have been circled. The mean images were calculated by using the number of subjects that have non-zero values for the voxel. Reproduced by kind permission of UK Biobank ©.

Supplementary Figure D: Mean number of voxels outside of the body mask in the deformed FF images.


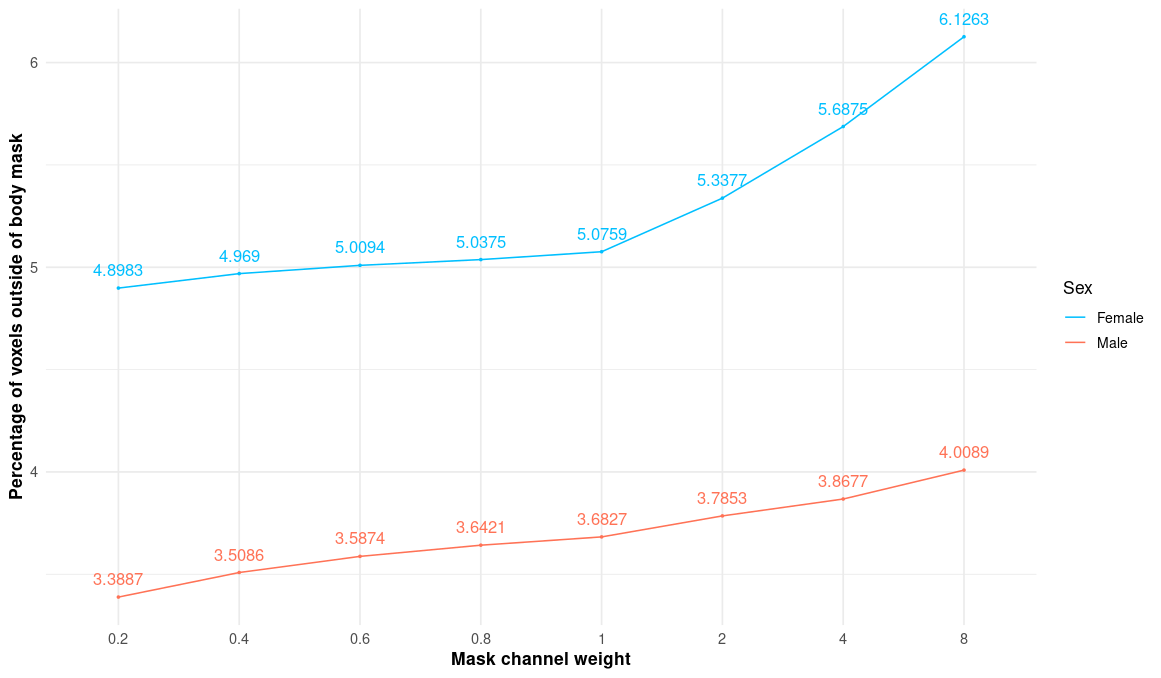


Mean number of voxels outside of the reference subject’s bodymask in mask-supported registration. Results are presented for the 200 subjects in the ablation subset, separately for males and females.

Supplementary Figure E: Per-voxel mean Jacobian Determinant fold rate maps for males and females with the intensity-based and mask-supported registrations.


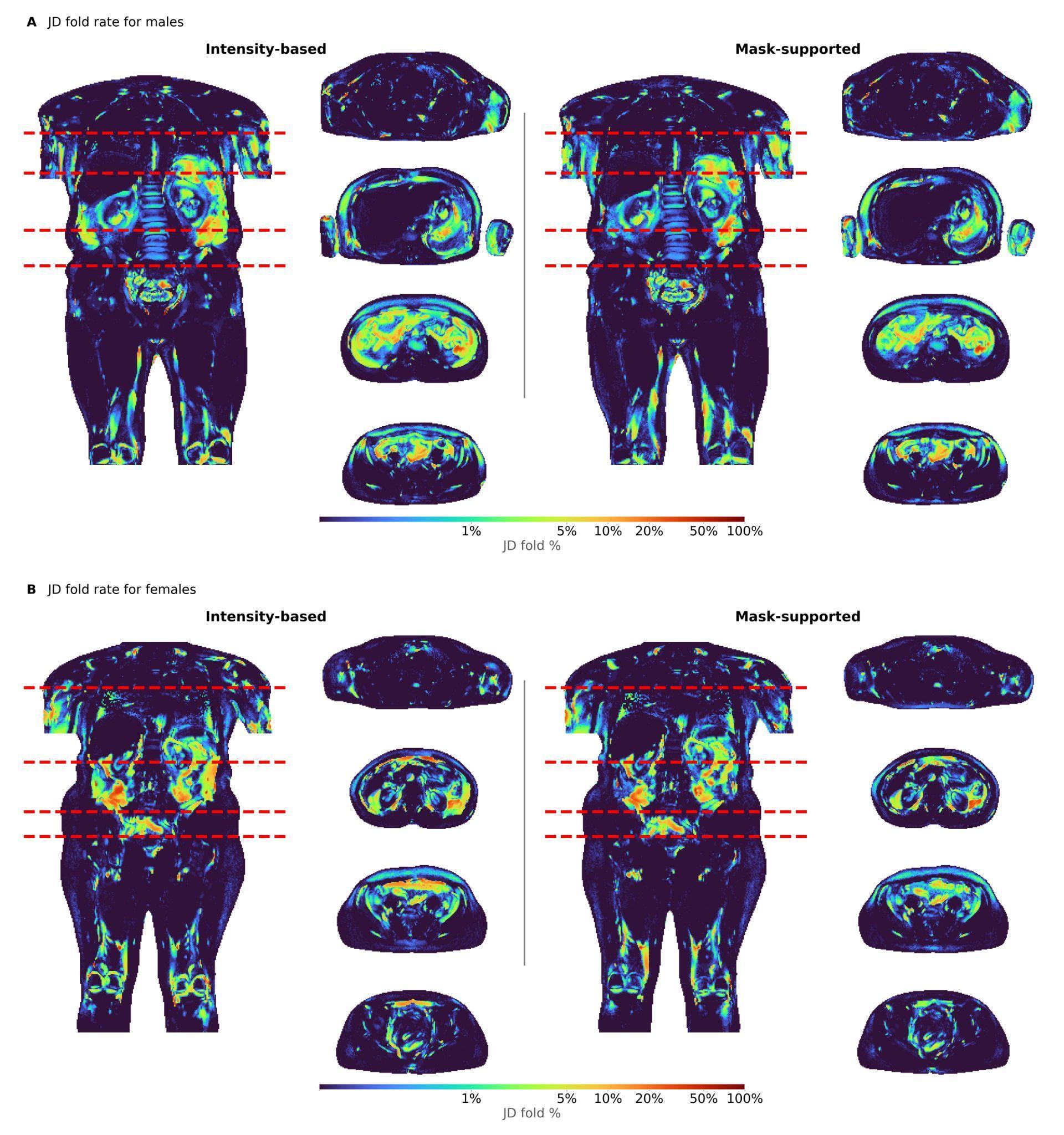


JD fold frequencies for the 2000 subjects per-sex and registration method. The colormap was scaled logarithmically for ease of visualisation. Reproduced by kind permission of UK Biobank ©.

Supplementary Table A: Demographic information of the 4000 subjects in this analysis and the reference subjects for each sex.

|  | **male** | | **female** | |
| --- | --- | --- | --- | --- |
|  | **cohort mean** | **reference subject** | **cohort mean** | **reference subject** |
| **age** | 64.7898 | 64.00 | 63.6863 | 63.59 |
| **body mass index (kg/m2)** | 27.1984 | 27.90 | 26.0847 | 22.70 |
| **waist circumference (cm)** | 94.7993 | 98 | 81.7322 | 73 |
| **hip circumference (cm)** | 102.8402 | 104 | 101.7272 | 98 |
| **body surface area (m2)** | 1.9978 | 1.97 | 1.7329 | 1.63 |

Supplementary Table B: Parameters used for the mask-supported registration.

| **Parameter** | **Value** |
| --- | --- |
| pyramid levels | 6 |
| pyramid stop level | 0 |
| block size | [12, 12, 12] |
| block energy epsilon | 1.00E-07 |
| max iteration count | 100 |
| step size | 0.5 |
| regularization scale | 1 |
| regularization exponent | 2 |
| regularization weight | 0.1 |
| image resampler | gaussian |
| cost function | ssd |
| update rule | additive |
| image normalization | TRUE |
| FF / WF image weight | 1 |
| binary mask weight | 0.6 |

Supplementary Table C: Mean dice scores of the 71 masks segmented with VIBESegmentator for the female and male cohorts for the intensity-based and mask-supported registrations. The statistical tests were done using pairwise Wilcoxon signed-rank tests with adjustment for multiple testing using Bonferroni correction. (na: not applicable, ns: not significant, *: p-value < 0.05, **: p-value < 0.01, ***: p-value < 0.001, ****: p-value < 0.0001)

|  | **male** | | | | **female** | | | |
| --- | --- | --- | --- | --- | --- | --- | --- | --- |
| **mask name** | **intensity-based** | **mask-supported** | **p.adj** | **significance** | **intensity-based** | **mask-supported** | **p.adj** | **significance** |
| **adrenal gland left** | 0.5632 | 0.5631 | 1.000 | ns | **0.3459** | 0.3380 | 0.000 | **** |
| **adrenal gland right** | 0.5567 | 0.5573 | 1.000 | ns | **0.3372** | 0.3292 | 0.000 | **** |
| **aorta** | 0.8467 | 0.8466 | 1.000 | ns | 0.7603 | **0.7649** | 0.000 | **** |
| **atrial appendage left** | **0.6055** | 0.5988 | 0.000 | **** | 0.3926 | 0.3924 | 1.000 | ns |
| **autochthon left** | 0.9179 | **0.9514** | 0.000 | **** | 0.9056 | **0.9397** | 0.000 | **** |
| **autochthon right** | 0.9097 | **0.9526** | 0.000 | **** | 0.9074 | **0.9468** | 0.000 | **** |
| **bone other** | 0.4908 | **0.6070** | 0.000 | **** | 0.6903 | **0.8310** | 0.000 | **** |
| **brachiocephalic trunk** | 0.6492 | **0.6817** | 0.000 | **** | 0.6156 | **0.6278** | 0.000 | **** |
| **brachiocephalic vein left** | 0.6300 | **0.6774** | 0.000 | **** | 0.6352 | **0.6653** | 0.000 | **** |
| **brachiocephalic vein right** | 0.6289 | **0.7202** | 0.000 | **** | 0.7224 | **0.7340** | 0.000 | **** |
| **clavicula left** | 0.6171 | **0.8372** | 0.000 | **** | 0.5898 | **0.8031** | 0.000 | **** |
| **clavicula right** | 0.4781 | **0.8131** | 0.000 | **** | 0.6494 | **0.8541** | 0.000 | **** |
| **common carotid artery left** | 0.5517 | **0.7214** | 0.000 | **** | 0.4978 | **0.6460** | 0.000 | **** |
| **common carotid artery right** | 0.4363 | **0.7618** | 0.000 | **** | 0.5734 | **0.7743** | 0.000 | **** |
| **costal cartilages** | 0.5480 | **0.7067** | 0.000 | **** | 0.6387 | **0.7535** | 0.000 | **** |
| **duodenum** | 0.6427 | **0.6610** | 0.000 | **** | 0.4560 | **0.4641** | 0.000 | **** |
| **esophagus** | 0.7012 | **0.7396** | 0.000 | **** | 0.6724 | **0.6969** | 0.000 | **** |
| **femur left** | 0.8903 | **0.9281** | 0.000 | **** | 0.8932 | **0.9656** | 0.000 | **** |
| **femur right** | 0.8879 | **0.9042** | 0.000 | **** | 0.8885 | **0.9602** | 0.000 | **** |
| **gallbladder** | 0.3550 | **0.3624** | 0.000 | **** | 0.2107 | **0.2199** | 0.000 | **** |
| **gluteus maximus left** | 0.9297 | **0.9651** | 0.000 | **** | 0.9325 | **0.9704** | 0.000 | **** |
| **gluteus maximus right** | 0.9438 | **0.9662** | 0.000 | **** | 0.9332 | **0.9717** | 0.000 | **** |
| **gluteus medius left** | 0.8926 | **0.9197** | 0.000 | **** | 0.8827 | **0.9155** | 0.000 | **** |
| **gluteus medius right** | 0.8999 | **0.9184** | 0.000 | **** | 0.8786 | **0.9124** | 0.000 | **** |
| **gluteus minimus left** | 0.8488 | **0.8678** | 0.000 | **** | 0.8213 | **0.8512** | 0.000 | **** |
| **gluteus minimus right** | 0.8451 | **0.8589** | 0.000 | **** | 0.8235 | **0.8569** | 0.000 | **** |
| **heart** | **0.8876** | 0.8876 | 0.000 | **** | **0.8961** | 0.8947 | 0.000 | **** |
| **hip left** | 0.8625 | **0.8991** | 0.000 | **** | 0.8562 | **0.9053** | 0.000 | **** |
| **hip right** | 0.8818 | **0.9015** | 0.000 | **** | 0.8423 | **0.8975** | 0.000 | **** |
| **humerus left** | 0.8061 | **0.9283** | 0.000 | **** | 0.7408 | **0.9209** | 0.000 | **** |
| **humerus right** | 0.5791 | **0.8026** | 0.000 | **** | 0.7917 | **0.9265** | 0.000 | **** |
| **iliac artery left** | 0.5562 | **0.5825** | 0.000 | **** | 0.5802 | **0.5986** | 0.000 | **** |
| **iliac artery right** | 0.5087 | **0.5375** | 0.000 | **** | 0.5517 | **0.5890** | 0.000 | **** |
| **iliac vena left** | 0.6843 | **0.6999** | 0.000 | **** | 0.6840 | **0.7021** | 0.000 | **** |
| **iliac vena right** | 0.6117 | **0.6239** | 0.000 | **** | 0.6444 | **0.6669** | 0.000 | **** |
| **iliopsoas left** | 0.8848 | **0.8999** | 0.000 | **** | 0.8444 | **0.8637** | 0.000 | **** |
| **iliopsoas right** | 0.8808 | **0.8916** | 0.000 | **** | 0.8163 | **0.8403** | 0.000 | **** |
| **inferior vena cava** | **0.7368** | 0.7339 | 0.000 | **** | 0.5960 | **0.5991** | 0.000 | *** |
| **inner fat** | 0.7302 | **0.7684** | 0.000 | **** | 0.6303 | **0.6910** | 0.000 | **** |
| **intestine** | 0.7371 | **0.7818** | 0.000 | **** | 0.7489 | **0.7963** | 0.000 | **** |
| **IVD** | 0.6588 | **0.6615** | 0.000 | **** | 0.5405 | **0.5463** | 0.000 | **** |
| **kidney left** | 0.8531 | **0.8636** | 0.000 | **** | **0.7547** | 0.7452 | 0.000 | **** |
| **kidney right** | 0.8708 | **0.8753** | 0.000 | **** | 0.6965 | 0.6969 | 1.000 | ns |
| **liver** | 0.9103 | **0.9143** | 0.000 | **** | 0.8677 | **0.8722** | 0.000 | **** |
| **lung lower lobe left** | 0.8317 | **0.8343** | 0.000 | **** | 0.8960 | **0.9018** | 0.000 | **** |
| **lung lower lobe right** | 0.8466 | **0.8468** | 0.000 | **** | 0.8796 | **0.8858** | 0.000 | **** |
| **lung middle lobe right** | 0.8250 | **0.8313** | 0.000 | **** | 0.8284 | **0.8369** | 0.000 | **** |
| **lung upper lobe left** | 0.8963 | **0.9092** | 0.000 | **** | 0.9038 | **0.9171** | 0.000 | **** |
| **lung upper lobe right** | 0.8813 | **0.8924** | 0.000 | **** | 0.9022 | **0.9121** | 0.000 | **** |
| **muscle** | 0.8867 | **0.9647** | 0.000 | **** | 0.8769 | **0.9658** | 0.000 | **** |
| **pancreas** | 0.6206 | **0.6298** | 0.000 | **** | 0.3445 | **0.3483** | 0.000 | *** |
| **portal vein and splenic vein** | 0.5514 | **0.5564** | 0.000 | **** | **0.2840** | 0.2775 | 0.000 | **** |
| **prostate** | 0.6197 | **0.6759** | 0.000 | **** | NA | NA | NA | NA |
| **pulmonary vein** | 0.6227 | 0.6217 | 1.000 | ns | **0.6088** | 0.6068 | 0.014 | * |
| **sacrum** | 0.8850 | **0.8963** | 0.000 | **** | 0.8374 | **0.8592** | 0.000 | **** |
| **scapula left** | 0.7844 | **0.9411** | 0.000 | **** | 0.6673 | **0.8947** | 0.000 | **** |
| **scapula right** | 0.6238 | **0.8781** | 0.000 | **** | 0.7050 | **0.9077** | 0.000 | **** |
| **spinal channel** | 0.7337 | **0.7500** | 0.000 | **** | 0.7138 | **0.7259** | 0.000 | **** |
| **spinal cord** | 0.6449 | **0.6762** | 0.000 | **** | 0.6507 | **0.6697** | 0.000 | **** |
| **spleen** | 0.7001 | **0.7064** | 0.000 | *** | 0.7005 | 0.6985 | 0.140 | ns |
| **sternum** | 0.6111 | **0.7286** | 0.000 | **** | 0.7636 | **0.8474** | 0.000 | **** |
| **stomach** | **0.6393** | 0.6361 | 0.003 | ** | 0.4958 | **0.5070** | 0.000 | **** |
| **subclavian artery left** | 0.5770 | **0.7504** | 0.000 | **** | 0.5259 | **0.6708** | 0.000 | **** |
| **subclavian artery right** | 0.5567 | **0.7990** | 0.000 | **** | 0.6519 | **0.7993** | 0.000 | **** |
| **subcutaneous fat** | 0.8795 | **0.9628** | 0.000 | **** | 0.9169 | **0.9756** | 0.000 | **** |
| **superior vena cava** | 0.7209 | **0.7281** | 0.000 | **** | 0.7825 | 0.7826 | 0.280 | ns |
| **thyroid gland** | 0.4876 | **0.8294** | 0.000 | **** | 0.5558 | **0.8086** | 0.000 | **** |
| **trachea** | 0.7283 | **0.7985** | 0.000 | **** | 0.7058 | **0.7455** | 0.000 | **** |
| **urinary bladder** | 0.6281 | **0.6433** | 0.000 | **** | 0.6975 | **0.7086** | 0.000 | **** |
| **vertebra body** | 0.7925 | **0.8001** | 0.000 | **** | 0.7165 | **0.7252** | 0.000 | **** |
| **vertebra posterior elements** | 0.6254 | **0.6495** | 0.000 | **** | 0.6145 | **0.6329** | 0.000 | **** |

Supplementary Table D: Mean dice scores of the 40 masks segmented with MRSegmentator for the female and male cohorts for the mask-supported and intensity-based registrations. The statistical tests were done using pairwise Wilcoxon signed-rank tests with adjustment for multiple testing using Bonferroni correction. (na: not applicable, ns: not significant, *: p-value < 0.05, **: p-value < 0.01, ***: p-value < 0.001, ****: p-value < 0.0001)

|  | **male** | | | | **female** | | | |
| --- | --- | --- | --- | --- | --- | --- | --- | --- |
| **mask name** | **intensity-based** | **mask-supported** | **p.adj** | **significance** | **intensity-based** | **mask-supported** | **p.adj** | **significance** |
| **aorta** | 0.8476 | **0.8476** | 0.3600 | ns | 0.7662 | **0.7701** | 0.0000 | **** |
| **colon** | 0.4827 | **0.5122** | 0.0000 | **** | 0.4050 | **0.4312** | 0.0000 | **** |
| **duodenum** | 0.5458 | **0.5616** | 0.0000 | **** | 0.4248 | **0.4316** | 0.0000 | **** |
| **esophagus** | 0.6512 | **0.6550** | 0.0000 | **** | 0.5673 | **0.5689** | 0.0031 | ** |
| **gallbladder** | 0.2457 | **0.2485** | 0.0024 | ** | 0.1119 | **0.1198** | 0.0000 | **** |
| **heart** | **0.8883** | 0.8880 | 0.3600 | ns | **0.8850** | 0.8835 | 0.0000 | **** |
| **inferior_vena_cava** | **0.6774** | 0.6760 | 0.0064 | ** | 0.4957 | **0.4965** | 1.0000 | ns |
| **left_adrenal_gland** | 0.5163 | **0.5170** | 1.0000 | ns | NA | NA | NA | NA |
| **left_autochthonous_muscle** | 0.9073 | **0.9102** | 0.0000 | **** | 0.8973 | **0.9017** | 0.0000 | **** |
| **left_femur** | 0.8686 | **0.8838** | 0.0000 | **** | 0.8844 | **0.9151** | 0.0000 | **** |
| **left_gluteus_maximus** | 0.9182 | **0.9234** | 0.0000 | **** | 0.9182 | **0.9247** | 0.0000 | **** |
| **left_gluteus_medius** | 0.8793 | **0.8878** | 0.0000 | **** | 0.8798 | **0.8910** | 0.0000 | **** |
| **left_gluteus_minimus** | 0.7949 | **0.8054** | 0.0000 | **** | 0.7623 | **0.7720** | 0.0000 | **** |
| **left_hip** | 0.8459 | **0.8676** | 0.0000 | **** | 0.8405 | **0.8706** | 0.0000 | **** |
| **left_iliac_artery** | 0.4833 | **0.4966** | 0.0000 | **** | 0.4802 | **0.4952** | 0.0000 | **** |
| **left_iliac_vena** | 0.6463 | **0.6530** | 0.0000 | **** | 0.6705 | **0.6830** | 0.0000 | **** |
| **left_iliopsoas_muscle** | 0.8837 | **0.8873** | 0.0000 | **** | 0.8444 | **0.8490** | 0.0000 | **** |
| **left_kidney** | 0.8567 | **0.8672** | 0.0000 | **** | **0.7560** | 0.7473 | 0.0000 | **** |
| **left_lung** | 0.9076 | **0.9097** | 0.0000 | **** | 0.9358 | **0.9390** | 0.0000 | **** |
| **liver** | 0.9069 | **0.9091** | 0.0000 | **** | 0.8661 | **0.8673** | 0.0000 | **** |
| **pancreas** | 0.5188 | **0.5279** | 0.0000 | **** | 0.2971 | **0.3001** | 0.0301 | * |
| **portal_vein_and_splenic_vein** | 0.3672 | **0.3713** | 0.0000 | **** | **0.1505** | 0.1479 | 0.0115 | * |
| **right_adrenal_gland** | 0.4968 | **0.4989** | 0.0035 | ** | **0.2215** | 0.2147 | 0.0000 | **** |
| **right_autochthonous_muscle** | 0.8925 | **0.8982** | 0.0000 | **** | 0.8817 | **0.8869** | 0.0000 | **** |
| **right_femur** | 0.8662 | **0.8798** | 0.0000 | **** | 0.8821 | **0.9110** | 0.0000 | **** |
| **right_gluteus_maximus** | 0.9331 | **0.9360** | 0.0000 | **** | 0.9177 | **0.9260** | 0.0000 | **** |
| **right_gluteus_medius** | 0.9006 | **0.9046** | 0.0000 | **** | 0.8767 | **0.8895** | 0.0000 | **** |
| **right_gluteus_minimus** | 0.8156 | **0.8237** | 0.0000 | **** | 0.7751 | **0.7881** | 0.0000 | **** |
| **right_hip** | 0.8632 | **0.8763** | 0.0000 | **** | 0.8257 | **0.8613** | 0.0000 | **** |
| **right_iliac_artery** | 0.3998 | **0.4013** | 0.0000 | **** | 0.4658 | **0.4856** | 0.0000 | **** |
| **right_iliac_vena** | 0.5399 | **0.5453** | 0.0000 | **** | 0.5766 | **0.5931** | 0.0000 | **** |
| **right_iliopsoas_muscle** | 0.8784 | **0.8819** | 0.0000 | **** | 0.8280 | **0.8372** | 0.0000 | **** |
| **right_kidney** | 0.8776 | **0.8824** | 0.0000 | **** | 0.6869 | **0.6877** | 1.0000 | ns |
| **right_lung** | 0.9208 | **0.9223** | 0.0000 | **** | 0.9380 | **0.9406** | 0.0000 | **** |
| **sacrum** | 0.8356 | **0.8482** | 0.0000 | **** | 0.7874 | **0.8101** | 0.0000 | **** |
| **small_bowel** | 0.5310 | **0.5639** | 0.0000 | **** | 0.4897 | **0.5325** | 0.0000 | **** |
| **spine** | 0.7827 | **0.7831** | 1.0000 | ns | 0.6902 | **0.6946** | 0.0000 | **** |
| **spleen** | 0.7116 | **0.7186** | 0.0007 | *** | **0.7062** | 0.7002 | 0.0000 | **** |
| **stomach** | **0.6022** | 0.5982 | 0.0000 | **** | 0.4507 | **0.4616** | 0.0000 | **** |
| **urinary_bladder** | 0.6120 | **0.6291** | 0.0000 | **** | 0.6550 | **0.6687** | 0.0000 | **** |

Supplementary Table E: Mean dice scores of the 50 masks segmented with TotalSegmentator for the female and male cohorts for the mask-supported and intensity-based registrations. The statistical tests were done using pairwise Wilcoxon signed-rank tests with adjustment for multiple testing using Bonferroni correction. (na: not applicable, ns: not significant, *: p-value < 0.05, **: p-value < 0.01, ***: p-value < 0.001, ****: p-value < 0.0001)

|  | **male** | | | | **female** | | | |
| --- | --- | --- | --- | --- | --- | --- | --- | --- |
| **mask name** | **intensity-based** | **mask-supported** | **p.adj** | **significance** | **intensity-based** | **mask-supported** | **p.adj** | **significance** |
| **adrenal_gland_left** | 0.4327 | **0.4339** | 1.0000 | ns | **0.1495** | 0.1384 | 0.0000 | **** |
| **adrenal_gland_right** | 0.4497 | **0.4516** | 0.2450 | ns | **0.2114** | 0.2034 | 0.0000 | **** |
| **aorta** | **0.8026** | 0.8005 | 0.0000 | **** | 0.7021 | **0.7062** | 0.0000 | **** |
| **autochthon_left** | 0.9039 | **0.9068** | 0.0000 | **** | 0.8885 | **0.8929** | 0.0000 | **** |
| **autochthon_right** | 0.8925 | **0.8994** | 0.0000 | **** | 0.8692 | **0.8762** | 0.0000 | **** |
| **clavicula_left** | 0.4268 | **0.5717** | 0.0000 | **** | 0.3863 | **0.5150** | 0.0000 | **** |
| **clavicula_right** | 0.2816 | **0.5050** | 0.0000 | **** | 0.4156 | **0.5156** | 0.0000 | **** |
| **colon** | 0.4465 | **0.4759** | 0.0000 | **** | 0.3477 | **0.3747** | 0.0000 | **** |
| **duodenum** | 0.4581 | **0.4763** | 0.0000 | **** | 0.2544 | **0.2574** | 0.0000 | **** |
| **esophagus** | 0.6122 | **0.6156** | 0.0000 | **** | 0.5426 | **0.5442** | 0.0480 | * |
| **femur_left** | 0.8563 | **0.8722** | 0.0000 | **** | 0.8665 | **0.8972** | 0.0000 | **** |
| **femur_right** | 0.8619 | **0.8758** | 0.0000 | **** | 0.8785 | **0.9038** | 0.0000 | **** |
| **gallbladder** | 0.2245 | **0.2269** | 0.0165 | * | 0.1133 | **0.1199** | 0.0000 | **** |
| **gluteus_maximus_left** | 0.9100 | **0.9145** | 0.0000 | **** | 0.9125 | **0.9189** | 0.0000 | **** |
| **gluteus_maximus_right** | 0.9149 | **0.9178** | 0.0000 | **** | 0.8996 | **0.9069** | 0.0000 | **** |
| **gluteus_medius_left** | 0.8194 | **0.8269** | 0.0000 | **** | 0.7500 | **0.7589** | 0.0000 | **** |
| **gluteus_medius_right** | 0.8406 | **0.8455** | 0.0000 | **** | 0.7338 | **0.7458** | 0.0000 | **** |
| **gluteus_minimus_left** | 0.7604 | **0.7703** | 0.0000 | **** | 0.7090 | **0.7179** | 0.0000 | **** |
| **gluteus_minimus_right** | 0.7546 | **0.7644** | 0.0000 | **** | 0.7065 | **0.7219** | 0.0000 | **** |
| **heart** | **0.8683** | 0.8678 | 1.0000 | ns | **0.8643** | 0.8632 | 0.0000 | **** |
| **hip_left** | 0.8075 | **0.8251** | 0.0000 | **** | 0.7815 | **0.8027** | 0.0000 | **** |
| **hip_right** | 0.8201 | **0.8312** | 0.0000 | **** | 0.7665 | **0.7971** | 0.0000 | **** |
| **humerus_left** | 0.7820 | **0.8263** | 0.0000 | **** | 0.7075 | **0.8010** | 0.0000 | **** |
| **humerus_right** | 0.5584 | **0.6805** | 0.0000 | **** | 0.7675 | **0.8181** | 0.0000 | **** |
| **iliac_artery_left** | 0.3945 | **0.4032** | 0.0000 | **** | 0.3190 | **0.3290** | 0.0000 | **** |
| **iliac_artery_right** | **0.3400** | 0.3359 | 0.0058 | ** | 0.3420 | **0.3546** | 0.0000 | **** |
| **iliac_vena_left** | 0.5512 | **0.5562** | 0.0000 | **** | 0.5486 | **0.5605** | 0.0000 | **** |
| **iliac_vena_right** | 0.4998 | **0.5024** | 1.0000 | ns | 0.4866 | **0.4995** | 0.0000 | **** |
| **iliopsoas_left** | 0.8518 | **0.8563** | 0.0000 | **** | 0.8147 | **0.8243** | 0.0000 | **** |
| **iliopsoas_right** | 0.8596 | **0.8666** | 0.0000 | **** | 0.8005 | **0.8114** | 0.0000 | **** |
| **inferior_vena_cava** | **0.5731** | 0.5723 | 1.0000 | ns | 0.2340 | **0.2391** | 0.0000 | **** |
| **intervertebral_discs** | 0.6163 | **0.6174** | 1.0000 | ns | 0.5007 | **0.5059** | 0.0000 | **** |
| **kidney_left** | 0.8121 | **0.8224** | 0.0000 | **** | **0.6815** | 0.6746 | 0.0000 | **** |
| **kidney_right** | 0.8250 | **0.8291** | 0.0000 | **** | **0.6057** | 0.6057 | 0.0270 | * |
| **liver** | 0.8903 | **0.8918** | 0.0000 | **** | **0.8500** | 0.8498 | 0.1440 | ns |
| **lung_left** | 0.9060 | **0.9086** | 0.0000 | **** | 0.9327 | **0.9362** | 0.0000 | **** |
| **lung_right** | 0.9212 | **0.9229** | 0.0000 | **** | 0.9415 | **0.9445** | 0.0000 | **** |
| **pancreas** | 0.3579 | **0.3653** | 0.0000 | **** | **0.0608** | 0.0588 | 0.0960 | ns |
| **portal_vein_and_splenic_vein** | 0.2906 | **0.2966** | 0.0000 | **** | **0.0750** | 0.0704 | 0.0000 | **** |
| **prostate** | 0.5778 | **0.6333** | 0.0000 | **** | NA | NA | NA | NA |
| **sacrum** | 0.7485 | **0.7583** | 0.0000 | **** | 0.6883 | **0.7094** | 0.0000 | **** |
| **scapula_left** | 0.6247 | **0.7053** | 0.0000 | **** | 0.5303 | **0.6530** | 0.0000 | **** |
| **scapula_right** | 0.4408 | **0.6134** | 0.0000 | **** | 0.5442 | **0.6597** | 0.0000 | **** |
| **small_bowel** | 0.3714 | **0.3959** | 0.0000 | **** | 0.2670 | **0.2944** | 0.0000 | **** |
| **spinal_cord** | 0.7596 | **0.7679** | 0.0000 | **** | 0.7417 | **0.7477** | 0.0000 | **** |
| **spleen** | 0.6905 | **0.6994** | 0.0000 | **** | **0.6711** | 0.6618 | 0.0000 | **** |
| **stomach** | **0.5803** | 0.5762 | 0.0000 | **** | 0.4302 | **0.4374** | 0.0000 | **** |
| **urinary_bladder** | 0.6214 | **0.6373** | 0.0000 | **** | 0.6358 | **0.6485** | 0.0000 | **** |
| **vertebrae** | 0.7402 | **0.7413** | 0.0112 | * | 0.6591 | **0.6640** | 0.0000 | **** |

Supplementary Table F: Mean dice scores of the 71 masks segmented with VIBESegmentator for the female and male cohorts for the intensity-based and uniGradICON registrations. The statistical tests were done using pairwise Wilcoxon signed-rank tests with adjustment for multiple testing using Bonferroni correction. (na: not applicable, ns: not significant, *: p-value < 0.05, **: p-value < 0.01, ***: p-value < 0.001, ****: p-value < 0.0001)

|  | **male** | | | | **female** | | | |
| --- | --- | --- | --- | --- | --- | --- | --- | --- |
| **mask name** | **intensity-based** | **uniGradICON** | **p.adj** | **significance** | **intensity-based** | **uniGradICON** | **p.adj** | **significance** |
| **adrenal gland left** | **0.4685** | 0.5632 | 0.000 | **** | **0.2205** | 0.3459 | 0.000 | **** |
| **adrenal gland right** | **0.4245** | 0.5567 | 0.000 | **** | **0.2770** | 0.3372 | 0.000 | **** |
| **aorta** | **0.7590** | 0.8467 | 0.000 | **** | 0.7076 | **0.7603** | 0.000 | **** |
| **atrial appendage left** | **0.4384** | 0.6055 | 0.000 | **** | **0.4123** | 0.3926 | 0.002 | ** |
| **autochthon left** | 0.9052 | **0.9179** | 0.000 | **** | 0.8980 | **0.9056** | 0.000 | **** |
| **autochthon right** | 0.9020 | **0.9097** | 0.000 | **** | 0.8912 | **0.9074** | 0.000 | **** |
| **bone other** | 0.3753 | **0.4908** | 0.000 | **** | 0.7032 | **0.6903** | 0.000 | **** |
| **brachiocephalic trunk** | 0.4827 | **0.6492** | 0.000 | **** | 0.5923 | **0.6156** | 0.000 | **** |
| **brachiocephalic vein left** | 0.4910 | **0.6300** | 0.000 | **** | 0.5751 | **0.6352** | 0.000 | **** |
| **brachiocephalic vein right** | 0.6327 | **0.6289** | 1.000 | ns | 0.6758 | **0.7224** | 0.000 | **** |
| **clavicula left** | 0.5312 | **0.6171** | 0.000 | **** | 0.5482 | **0.5898** | 0.000 | **** |
| **clavicula right** | 0.5128 | **0.4781** | 0.000 | **** | 0.5609 | **0.6494** | 0.000 | **** |
| **common carotid artery left** | 0.4333 | **0.5517** | 0.000 | **** | 0.5320 | **0.4978** | 0.000 | **** |
| **common carotid artery right** | 0.4848 | **0.4363** | 0.000 | **** | 0.4918 | **0.5734** | 0.000 | **** |
| **costal cartilages** | 0.4748 | **0.5480** | 0.000 | **** | 0.5369 | **0.6387** | 0.000 | **** |
| **duodenum** | 0.6356 | **0.6427** | 0.000 | **** | 0.4216 | **0.4560** | 0.000 | **** |
| **esophagus** | 0.6312 | **0.7012** | 0.000 | **** | 0.6243 | **0.6724** | 0.000 | **** |
| **femur left** | 0.8718 | **0.8903** | 0.000 | **** | 0.8885 | **0.8932** | 0.630 | ns |
| **femur right** | 0.8761 | **0.8879** | 0.000 | **** | 0.8766 | **0.8885** | 0.000 | **** |
| **gallbladder** | 0.2686 | **0.3550** | 0.000 | **** | 0.2071 | **0.2107** | 1.000 | ns |
| **gluteus maximus left** | 0.9227 | **0.9297** | 0.000 | **** | 0.9288 | **0.9325** | 0.000 | **** |
| **gluteus maximus right** | 0.9350 | **0.9438** | 0.000 | **** | 0.9199 | **0.9332** | 0.000 | **** |
| **gluteus medius left** | 0.8820 | **0.8926** | 0.000 | **** | 0.8501 | **0.8827** | 0.000 | **** |
| **gluteus medius right** | 0.8893 | **0.8999** | 0.000 | **** | 0.8489 | **0.8786** | 0.000 | **** |
| **gluteus minimus left** | 0.8430 | **0.8488** | 0.000 | **** | 0.7947 | **0.8213** | 0.000 | **** |
| **gluteus minimus right** | 0.8303 | **0.8451** | 0.000 | **** | 0.8101 | **0.8235** | 0.000 | **** |
| **heart** | **0.8419** | 0.8876 | 0.000 | **** | **0.8529** | 0.8961 | 0.000 | **** |
| **hip left** | 0.8300 | **0.8625** | 0.000 | **** | 0.8093 | **0.8562** | 0.000 | **** |
| **hip right** | 0.8474 | **0.8818** | 0.000 | **** | 0.7980 | **0.8423** | 0.000 | **** |
| **humerus left** | 0.7278 | **0.8061** | 0.000 | **** | 0.7321 | **0.7408** | 1.000 | ns |
| **humerus right** | 0.5475 | **0.5791** | 0.000 | **** | 0.7313 | **0.7917** | 0.000 | **** |
| **iliac artery left** | 0.4732 | **0.5562** | 0.000 | **** | 0.5016 | **0.5802** | 0.000 | **** |
| **iliac artery right** | 0.4021 | **0.5087** | 0.000 | **** | 0.4363 | **0.5517** | 0.000 | **** |
| **iliac vena left** | 0.6088 | **0.6843** | 0.000 | **** | 0.5725 | **0.6840** | 0.000 | **** |
| **iliac vena right** | 0.5065 | **0.6117** | 0.000 | **** | 0.5049 | **0.6444** | 0.000 | **** |
| **iliopsoas left** | 0.8656 | **0.8848** | 0.000 | **** | 0.8256 | **0.8444** | 0.000 | **** |
| **iliopsoas right** | 0.8637 | **0.8808** | 0.000 | **** | 0.8201 | **0.8163** | 0.140 | ns |
| **inferior vena cava** | **0.6880** | 0.7368 | 0.000 | **** | 0.5476 | **0.5960** | 0.000 | **** |
| **inner fat** | 0.6946 | **0.7302** | 0.000 | **** | 0.5651 | **0.6303** | 0.000 | **** |
| **intestine** | 0.7230 | **0.7371** | 0.000 | **** | 0.7480 | **0.7489** | 0.000 | **** |
| **IVD** | 0.4367 | **0.6588** | 0.000 | **** | 0.3417 | **0.5405** | 0.000 | **** |
| **kidney left** | 0.8364 | **0.8531** | 0.000 | **** | **0.7234** | 0.7547 | 0.000 | **** |
| **kidney right** | 0.8317 | **0.8708** | 0.000 | **** | **0.7893** | 0.6965 | 0.000 | **** |
| **liver** | 0.8865 | **0.9103** | 0.000 | **** | 0.8432 | **0.8677** | 0.000 | **** |
| **lung lower lobe left** | 0.8652 | **0.8317** | 0.000 | **** | 0.8894 | **0.8960** | 0.000 | **** |
| **lung lower lobe right** | **0.8976** | 0.8466 | 0.000 | **** | 0.9052 | **0.8796** | 0.000 | **** |
| **lung middle lobe right** | 0.8350 | **0.8250** | 0.000 | **** | 0.8304 | **0.8284** | 1.000 | ns |
| **lung upper lobe left** | 0.9001 | **0.8963** | 0.000 | **** | 0.9070 | **0.9038** | 0.000 | **** |
| **lung upper lobe right** | 0.8959 | **0.8813** | 0.000 | **** | 0.9093 | **0.9022** | 0.000 | **** |
| **muscle** | 0.8745 | **0.8867** | 0.000 | **** | 0.8752 | **0.8769** | 1.000 | ns |
| **pancreas** | 0.6007 | **0.6206** | 0.000 | **** | **0.4185** | 0.3445 | 0.000 | **** |
| **portal vein and splenic vein** | 0.4241 | **0.5514** | 0.000 | **** | **0.4129** | 0.2840 | 0.000 | **** |
| **prostate** | 0.6367 | **0.6197** | 0.000 | **** | NA | NA | NA | NA |
| **pulmonary vein** | **0.6876** | 0.6227 | 0.000 | **** | **0.7025** | 0.6088 | 0.000 | **** |
| **sacrum** | 0.8739 | **0.8850** | 0.000 | **** | 0.7887 | **0.8374** | 0.000 | **** |
| **scapula left** | 0.7259 | **0.7844** | 0.000 | **** | 0.6719 | **0.6673** | 0.004 | ** |
| **scapula right** | 0.6176 | **0.6238** | 0.000 | **** | 0.6568 | **0.7050** | 0.000 | **** |
| **spinal channel** | 0.7190 | **0.7337** | 0.000 | **** | 0.6893 | **0.7138** | 0.000 | **** |
| **spinal cord** | 0.5971 | **0.6449** | 0.000 | **** | 0.6005 | **0.6507** | 0.000 | **** |
| **spleen** | **0.7139** | 0.7001 | 0.000 | **** | **0.5879** | 0.7005 | 0.000 | **** |
| **sternum** | 0.5649 | **0.6111** | 0.000 | **** | 0.6931 | **0.7636** | 0.000 | **** |
| **stomach** | **0.6634** | 0.6393 | 0.000 | **** | 0.4474 | **0.4958** | 0.000 | **** |
| **subclavian artery left** | 0.4611 | **0.5770** | 0.000 | **** | 0.5649 | **0.5259** | 0.000 | **** |
| **subclavian artery right** | 0.4900 | **0.5567** | 0.000 | **** | 0.5691 | **0.6519** | 0.000 | **** |
| **subcutaneous fat** | 0.8887 | **0.8795** | 0.000 | **** | 0.9220 | **0.9169** | 0.000 | **** |
| **superior vena cava** | 0.7400 | **0.7209** | 0.000 | **** | **0.7478** | 0.7825 | 0.000 | **** |
| **thyroid gland** | 0.4680 | **0.4876** | 0.000 | **** | 0.4482 | **0.5558** | 0.000 | **** |
| **trachea** | 0.7286 | **0.7283** | 0.923 | ns | 0.7237 | **0.7058** | 0.000 | **** |
| **urinary bladder** | 0.7656 | **0.6281** | 0.000 | **** | 0.8259 | **0.6975** | 0.000 | **** |
| **vertebra body** | 0.7168 | **0.7925** | 0.000 | **** | 0.6575 | **0.7165** | 0.000 | **** |
| **vertebra posterior elements** | 0.6425 | **0.6254** | 0.000 | **** | 0.5940 | **0.6145** | 0.000 | **** |

Supplementary Table G: Mean dice scores of the 71 masks segmented with VIBESegmentator for the female and male cohorts for the mask-supported and uniGradICON registrations. The statistical tests were done using pairwiseWilcoxon signed-rank tests with adjustment for multiple testing using Bonferroni correction. (na: not applicable, ns: not significant, *: p-value < 0.05, **: p-value < 0.01, ***: p-value < 0.001, ****: p-value < 0.0001)

|  | **male** | | | | **female** | | | |
| --- | --- | --- | --- | --- | --- | --- | --- | --- |
| **mask name** | **uniGradICON** | **mask-supported** | **p.adj** | **significance** | **uniGradICON** | **mask-supported** | **p.adj** | **significance** |
| **adrenal gland left** | 0.4685 | **0.5631** | 0.000 | **** | 0.2205 | **0.3380** | 0.000 | **** |
| **adrenal gland right** | 0.4245 | **0.5573** | 0.000 | **** | 0.2770 | **0.3292** | 0.000 | **** |
| **aorta** | 0.7590 | **0.8466** | 0.000 | **** | 0.7076 | **0.7649** | 0.000 | **** |
| **atrial appendage left** | 0.4384 | **0.5988** | 0.000 | **** | **0.4123** | 0.3924 | 0.002 | ** |
| **autochthon left** | 0.9052 | **0.9514** | 0.000 | **** | 0.8980 | **0.9397** | 0.000 | **** |
| **autochthon right** | 0.9020 | **0.9526** | 0.000 | **** | 0.8912 | **0.9468** | 0.000 | **** |
| **bone other** | 0.3753 | **0.6070** | 0.000 | **** | 0.7032 | **0.8310** | 0.000 | **** |
| **brachiocephalic trunk** | 0.4827 | **0.6817** | 0.000 | **** | 0.5923 | **0.6278** | 0.000 | **** |
| **brachiocephalic vein left** | 0.4910 | **0.6774** | 0.000 | **** | 0.5751 | **0.6653** | 0.000 | **** |
| **brachiocephalic vein right** | 0.6327 | **0.7202** | 0.000 | **** | 0.6758 | **0.7340** | 0.000 | **** |
| **clavicula left** | 0.5312 | **0.8372** | 0.000 | **** | 0.5482 | **0.8031** | 0.000 | **** |
| **clavicula right** | 0.5128 | **0.8131** | 0.000 | **** | 0.5609 | **0.8541** | 0.000 | **** |
| **common carotid artery left** | 0.4333 | **0.7214** | 0.000 | **** | 0.5320 | **0.6460** | 0.000 | **** |
| **common carotid artery right** | 0.4848 | **0.7618** | 0.000 | **** | 0.4918 | **0.7743** | 0.000 | **** |
| **costal cartilages** | 0.4748 | **0.7067** | 0.000 | **** | 0.5369 | **0.7535** | 0.000 | **** |
| **duodenum** | 0.6356 | **0.6610** | 0.000 | **** | 0.4216 | **0.4641** | 0.000 | **** |
| **esophagus** | 0.6312 | **0.7396** | 0.000 | **** | 0.6243 | **0.6969** | 0.000 | **** |
| **femur left** | 0.8718 | **0.9281** | 0.000 | **** | 0.8885 | **0.9656** | 0.000 | **** |
| **femur right** | 0.8761 | **0.9042** | 0.000 | **** | 0.8766 | **0.9602** | 0.000 | **** |
| **gallbladder** | 0.2686 | **0.3624** | 0.000 | **** | 0.2071 | **0.2199** | 0.000 | *** |
| **gluteus maximus left** | 0.9227 | **0.9651** | 0.000 | **** | 0.9288 | **0.9704** | 0.000 | **** |
| **gluteus maximus right** | 0.9350 | **0.9662** | 0.000 | **** | 0.9199 | **0.9717** | 0.000 | **** |
| **gluteus medius left** | 0.8820 | **0.9197** | 0.000 | **** | 0.8501 | **0.9155** | 0.000 | **** |
| **gluteus medius right** | 0.8893 | **0.9184** | 0.000 | **** | 0.8489 | **0.9124** | 0.000 | **** |
| **gluteus minimus left** | 0.8430 | **0.8678** | 0.000 | **** | 0.7947 | **0.8512** | 0.000 | **** |
| **gluteus minimus right** | 0.8303 | **0.8589** | 0.000 | **** | 0.8101 | **0.8569** | 0.000 | **** |
| **heart** | 0.8419 | **0.8876** | 0.000 | **** | 0.8529 | **0.8947** | 0.000 | **** |
| **hip left** | 0.8300 | **0.8991** | 0.000 | **** | 0.8093 | **0.9053** | 0.000 | **** |
| **hip right** | 0.8474 | **0.9015** | 0.000 | **** | 0.7980 | **0.8975** | 0.000 | **** |
| **humerus left** | 0.7278 | **0.9283** | 0.000 | **** | 0.7321 | **0.9209** | 0.000 | **** |
| **humerus right** | 0.5475 | **0.8026** | 0.000 | **** | 0.7313 | **0.9265** | 0.000 | **** |
| **iliac artery left** | 0.4732 | **0.5825** | 0.000 | **** | 0.5016 | **0.5986** | 0.000 | **** |
| **iliac artery right** | 0.4021 | **0.5375** | 0.000 | **** | 0.4363 | **0.5890** | 0.000 | **** |
| **iliac vena left** | 0.6088 | **0.6999** | 0.000 | **** | 0.5725 | **0.7021** | 0.000 | **** |
| **iliac vena right** | 0.5065 | **0.6239** | 0.000 | **** | 0.5049 | **0.6669** | 0.000 | **** |
| **iliopsoas left** | 0.8656 | **0.8999** | 0.000 | **** | 0.8256 | **0.8637** | 0.000 | **** |
| **iliopsoas right** | 0.8637 | **0.8916** | 0.000 | **** | 0.8201 | **0.8403** | 0.000 | **** |
| **inferior vena cava** | 0.6880 | **0.7339** | 0.000 | **** | 0.5476 | **0.5991** | 0.000 | **** |
| **inner fat** | 0.6946 | **0.7684** | 0.000 | **** | 0.5651 | **0.6910** | 0.000 | **** |
| **intestine** | 0.7230 | **0.7818** | 0.000 | **** | 0.7480 | **0.7963** | 0.000 | **** |
| **IVD** | 0.4367 | **0.6615** | 0.000 | **** | 0.3417 | **0.5463** | 0.000 | **** |
| **kidney left** | 0.8364 | **0.8636** | 0.000 | **** | 0.7234 | **0.7452** | 0.000 | **** |
| **kidney right** | 0.8317 | **0.8753** | 0.000 | **** | **0.7893** | 0.6969 | 0.000 | **** |
| **liver** | 0.8865 | **0.9143** | 0.000 | **** | 0.8432 | **0.8722** | 0.000 | **** |
| **lung lower lobe left** | **0.8652** | 0.8343 | 0.000 | **** | 0.8894 | **0.9018** | 0.000 | **** |
| **lung lower lobe right** | **0.8976** | 0.8468 | 0.000 | **** | **0.9052** | 0.8858 | 0.000 | **** |
| **lung middle lobe right** | **0.8350** | 0.8313 | 0.000 | **** | 0.8304 | **0.8369** | 0.000 | **** |
| **lung upper lobe left** | 0.9001 | **0.9092** | 0.000 | **** | 0.9070 | **0.9171** | 0.000 | **** |
| **lung upper lobe right** | **0.8959** | 0.8924 | 0.000 | **** | 0.9093 | **0.9121** | 0.000 | **** |
| **muscle** | 0.8745 | **0.9647** | 0.000 | **** | 0.8752 | **0.9658** | 0.000 | **** |
| **pancreas** | 0.6007 | **0.6298** | 0.000 | **** | **0.4185** | 0.3483 | 0.000 | **** |
| **portal vein and splenic vein** | 0.4241 | **0.5564** | 0.000 | **** | **0.4129** | 0.2775 | 0.000 | **** |
| **prostate** | 0.6367 | **0.6759** | 0.000 | **** | NA | NA | NA | NA |
| **pulmonary vein** | **0.6876** | 0.6217 | 0.000 | **** | **0.7025** | 0.6068 | 0.000 | **** |
| **sacrum** | 0.8739 | **0.8963** | 0.000 | **** | 0.7887 | **0.8592** | 0.000 | **** |
| **scapula left** | 0.7259 | **0.9411** | 0.000 | **** | 0.6719 | **0.8947** | 0.000 | **** |
| **scapula right** | 0.6176 | **0.8781** | 0.000 | **** | 0.6568 | **0.9077** | 0.000 | **** |
| **spinal channel** | 0.7190 | **0.7500** | 0.000 | **** | 0.6893 | **0.7259** | 0.000 | **** |
| **spinal cord** | 0.5971 | **0.6762** | 0.000 | **** | 0.6005 | **0.6697** | 0.000 | **** |
| **spleen** | **0.7139** | 0.7064 | 0.000 | **** | 0.5879 | **0.6985** | 0.000 | **** |
| **sternum** | 0.5649 | **0.7286** | 0.000 | **** | 0.6931 | **0.8474** | 0.000 | **** |
| **stomach** | **0.6634** | 0.6361 | 0.000 | **** | 0.4474 | **0.5070** | 0.000 | **** |
| **subclavian artery left** | 0.4611 | **0.7504** | 0.000 | **** | 0.5649 | **0.6708** | 0.000 | **** |
| **subclavian artery right** | 0.4900 | **0.7990** | 0.000 | **** | 0.5691 | **0.7993** | 0.000 | **** |
| **subcutaneous fat** | 0.8887 | **0.9628** | 0.000 | **** | 0.9220 | **0.9756** | 0.000 | **** |
| **superior vena cava** | **0.7400** | 0.7281 | 0.000 | **** | 0.7478 | **0.7826** | 0.000 | **** |
| **thyroid gland** | 0.4680 | **0.8294** | 0.000 | **** | 0.4482 | **0.8086** | 0.000 | **** |
| **trachea** | 0.7286 | **0.7985** | 0.000 | **** | 0.7237 | **0.7455** | 0.000 | **** |
| **urinary bladder** | **0.7656** | 0.6433 | 0.000 | **** | **0.8259** | 0.7086 | 0.000 | **** |
| **vertebra body** | 0.7168 | **0.8001** | 0.000 | **** | 0.6575 | **0.7252** | 0.000 | **** |
| **vertebra posterior elements** | 0.6425 | **0.6495** | 0.000 | **** | 0.5940 | **0.6329** | 0.000 | **** |

Supplementary Table H: Mean dice scores of the 40 masks segmented with MRSegmentator for the female and male cohorts for the mask-supported and uniGradICON registrations. The statistical tests were done using pairwise Wilcoxon signed-rank tests with adjustment for multiple testing using Bonferroni correction. (na: not applicable, ns: not significant, *: p-value < 0.05, **: p-value < 0.01, ***: p-value < 0.001, ****: p-value < 0.0001)

|  | **male** | | | | **female** | | | |
| --- | --- | --- | --- | --- | --- | --- | --- | --- |
| **mask name** | **uniGradICON** | **mask-supported** | **p.adj** | **significance** | **uniGradICON** | **mask-supported** | **p.adj** | **significance** |
| **aorta** | 0.7261 | **0.8476** | 0.0000 | **** | 0.6693 | **0.7701** | 0.0000 | **** |
| **colon** | 0.4807 | **0.5122** | 0.0000 | **** | **0.4404** | 0.4312 | 0.0000 | **** |
| **duodenum** | **0.5719** | 0.5616 | 0.0149 | * | 0.3257 | **0.4316** | 0.0000 | **** |
| **esophagus** | 0.5351 | **0.6550** | 0.0000 | **** | 0.5194 | **0.5689** | 0.0000 | **** |
| **gallbladder** | 0.2358 | **0.2485** | 0.0050 | ** | **0.1671** | 0.1198 | 0.0000 | **** |
| **heart** | 0.8271 | **0.8880** | 0.0000 | **** | 0.8424 | **0.8835** | 0.0000 | **** |
| **inferior_vena_cava** | 0.6118 | **0.6760** | 0.0000 | **** | 0.3798 | **0.4965** | 0.0000 | **** |
| **left_adrenal_gland** | 0.3692 | **0.5170** | 0.0000 | **** | NA | NA | NA | NA |
| **left_autochthonous_muscle** | 0.9055 | **0.9102** | 0.0000 | **** | 0.8944 | **0.9017** | 0.0000 | **** |
| **left_femur** | 0.8516 | **0.8838** | 0.0000 | **** | 0.8775 | **0.9151** | 0.0000 | **** |
| **left_gluteus_maximus** | 0.9144 | **0.9234** | 0.0000 | **** | 0.9162 | **0.9247** | 0.0000 | **** |
| **left_gluteus_medius** | 0.8795 | **0.8878** | 0.0000 | **** | 0.8562 | **0.8910** | 0.0000 | **** |
| **left_gluteus_minimus** | 0.7912 | **0.8054** | 0.0000 | **** | 0.7315 | **0.7720** | 0.0000 | **** |
| **left_hip** | 0.8061 | **0.8676** | 0.0000 | **** | 0.7862 | **0.8706** | 0.0000 | **** |
| **left_iliac_artery** | 0.3251 | **0.4966** | 0.0000 | **** | 0.3305 | **0.4952** | 0.0000 | **** |
| **left_iliac_vena** | 0.5484 | **0.6530** | 0.0000 | **** | 0.5197 | **0.6830** | 0.0000 | **** |
| **left_iliopsoas_muscle** | 0.8550 | **0.8873** | 0.0000 | **** | 0.8144 | **0.8490** | 0.0000 | **** |
| **left_kidney** | 0.8540 | **0.8672** | 0.0000 | **** | 0.7224 | **0.7473** | 0.0000 | **** |
| **left_lung** | **0.9446** | 0.9097 | 0.0000 | **** | **0.9512** | 0.9390 | 0.0000 | **** |
| **liver** | 0.8937 | **0.9091** | 0.0000 | **** | 0.8533 | **0.8673** | 0.0000 | **** |
| **pancreas** | 0.5126 | **0.5279** | 0.0000 | **** | 0.2786 | **0.3001** | 0.0000 | **** |
| **portal_vein_and_splenic_vein** | 0.2400 | **0.3713** | 0.0000 | **** | **0.1907** | 0.1479 | 0.0000 | **** |
| **right_adrenal_gland** | 0.4123 | **0.4989** | 0.0000 | **** | 0.1366 | **0.2147** | 0.0000 | **** |
| **right_autochthonous_muscle** | 0.8969 | **0.8982** | 0.0000 | **** | **0.8927** | 0.8869 | 0.0000 | **** |
| **right_femur** | 0.8639 | **0.8798** | 0.0000 | **** | 0.8727 | **0.9110** | 0.0000 | **** |
| **right_gluteus_maximus** | 0.9294 | **0.9360** | 0.0000 | **** | 0.9084 | **0.9260** | 0.0000 | **** |
| **right_gluteus_medius** | 0.8824 | **0.9046** | 0.0000 | **** | 0.8523 | **0.8895** | 0.0000 | **** |
| **right_gluteus_minimus** | 0.8001 | **0.8237** | 0.0000 | **** | 0.7661 | **0.7881** | 0.0000 | **** |
| **right_hip** | 0.8210 | **0.8763** | 0.0000 | **** | 0.7684 | **0.8613** | 0.0000 | **** |
| **right_iliac_artery** | 0.2461 | **0.4013** | 0.0000 | **** | 0.2639 | **0.4856** | 0.0000 | **** |
| **right_iliac_vena** | 0.3766 | **0.5453** | 0.0000 | **** | 0.3789 | **0.5931** | 0.0000 | **** |
| **right_iliopsoas_muscle** | 0.8571 | **0.8819** | 0.0000 | **** | 0.8061 | **0.8372** | 0.0000 | **** |
| **right_kidney** | 0.8447 | **0.8824** | 0.0000 | **** | **0.7831** | 0.6877 | 0.0000 | **** |
| **right_lung** | **0.9570** | 0.9223 | 0.0000 | **** | **0.9624** | 0.9406 | 0.0000 | **** |
| **sacrum** | 0.8160 | **0.8482** | 0.0000 | **** | 0.7061 | **0.8101** | 0.0000 | **** |
| **small_bowel** | 0.5080 | **0.5639** | 0.0000 | **** | 0.5015 | **0.5325** | 0.0000 | **** |
| **spine** | 0.6959 | **0.7831** | 0.0000 | **** | 0.6142 | **0.6946** | 0.0000 | **** |
| **spleen** | 0.7167 | **0.7186** | 0.0000 | **** | 0.5924 | **0.7002** | 0.0000 | **** |
| **stomach** | **0.6591** | 0.5982 | 0.0000 | **** | 0.4224 | **0.4616** | 0.0000 | **** |
| **urinary_bladder** | **0.7567** | 0.6291 | 0.0000 | **** | **0.8065** | 0.6687 | 0.0000 | **** |

Supplementary Table I: Mean dice scores of the 50 masks segmented with TotalSegmentator for the female and male cohorts for the mask-supported and uniGradICON registrations. The statistical tests were done using pairwise Wilcoxon signed-rank tests with adjustment for multiple testing using Bonferroni correction. (na: not applicable, ns: not significant, *: p-value < 0.05, **: p-value < 0.01, ***: p-value < 0.001, ****: p-value < 0.0001)

|  | **male** | | | | **female** | | | |
| --- | --- | --- | --- | --- | --- | --- | --- | --- |
| **mask name** | **uniGradICON** | **mask-supported** | **p.adj** | **significance** | **uniGradICON** | **mask-supported** | **p.adj** | **significance** |
| **adrenal_gland_left** | 0.3529 | **0.4339** | 0.0000 | **** | 0.0565 | **0.1384** | 0.0000 | **** |
| **adrenal_gland_right** | 0.3992 | **0.4516** | 0.0000 | **** | 0.1610 | **0.2034** | 0.0000 | **** |
| **aorta** | 0.6532 | **0.8005** | 0.0000 | **** | 0.5924 | **0.7062** | 0.0000 | **** |
| **autochthon_left** | 0.9013 | **0.9068** | 0.0000 | **** | 0.8856 | **0.8929** | 0.0000 | **** |
| **autochthon_right** | 0.8912 | **0.8994** | 0.0000 | **** | **0.8785** | 0.8762 | 0.8640 | ns |
| **clavicula_left** | 0.3037 | **0.5717** | 0.0000 | **** | 0.2898 | **0.5150** | 0.0000 | **** |
| **clavicula_right** | 0.2494 | **0.5050** | 0.0000 | **** | 0.2707 | **0.5156** | 0.0000 | **** |
| **colon** | 0.4580 | **0.4759** | 0.0000 | **** | **0.4092** | 0.3747 | 0.0000 | **** |
| **duodenum** | **0.5184** | 0.4763 | 0.0000 | **** | 0.1721 | **0.2574** | 0.0000 | **** |
| **esophagus** | 0.5286 | **0.6156** | 0.0000 | **** | 0.5081 | **0.5442** | 0.0000 | **** |
| **femur_left** | 0.8338 | **0.8722** | 0.0000 | **** | 0.8570 | **0.8972** | 0.0000 | **** |
| **femur_right** | 0.8550 | **0.8758** | 0.0000 | **** | 0.8633 | **0.9038** | 0.0000 | **** |
| **gallbladder** | 0.2208 | **0.2269** | 0.6860 | ns | **0.1804** | 0.1199 | 0.0000 | **** |
| **gluteus_maximus_left** | 0.9064 | **0.9145** | 0.0000 | **** | 0.9117 | **0.9189** | 0.0000 | **** |
| **gluteus_maximus_right** | 0.9096 | **0.9178** | 0.0000 | **** | 0.8892 | **0.9069** | 0.0000 | **** |
| **gluteus_medius_left** | 0.8192 | **0.8269** | 0.0000 | **** | 0.7301 | **0.7589** | 0.0000 | **** |
| **gluteus_medius_right** | 0.8254 | **0.8455** | 0.0000 | **** | 0.7025 | **0.7458** | 0.0000 | **** |
| **gluteus_minimus_left** | 0.7464 | **0.7703** | 0.0000 | **** | 0.6695 | **0.7179** | 0.0000 | **** |
| **gluteus_minimus_right** | 0.7467 | **0.7644** | 0.0000 | **** | 0.6963 | **0.7219** | 0.0000 | **** |
| **heart** | 0.8237 | **0.8678** | 0.0000 | **** | 0.8144 | **0.8632** | 0.0000 | **** |
| **hip_left** | 0.7432 | **0.8251** | 0.0000 | **** | 0.7176 | **0.8027** | 0.0000 | **** |
| **hip_right** | 0.7546 | **0.8312** | 0.0000 | **** | 0.6828 | **0.7971** | 0.0000 | **** |
| **humerus_left** | 0.7321 | **0.8263** | 0.0000 | **** | 0.7072 | **0.8010** | 0.0000 | **** |
| **humerus_right** | 0.5148 | **0.6805** | 0.0000 | **** | 0.6983 | **0.8181** | 0.0000 | **** |
| **iliac_artery_left** | 0.2806 | **0.4032** | 0.0000 | **** | 0.2461 | **0.3290** | 0.0000 | **** |
| **iliac_artery_right** | 0.2182 | **0.3359** | 0.0000 | **** | 0.1958 | **0.3546** | 0.0000 | **** |
| **iliac_vena_left** | 0.4895 | **0.5562** | 0.0000 | **** | 0.4438 | **0.5605** | 0.0000 | **** |
| **iliac_vena_right** | 0.3448 | **0.5024** | 0.0000 | **** | 0.3368 | **0.4995** | 0.0000 | **** |
| **iliopsoas_left** | 0.8274 | **0.8563** | 0.0000 | **** | 0.7907 | **0.8243** | 0.0000 | **** |
| **iliopsoas_right** | 0.8335 | **0.8666** | 0.0000 | **** | 0.7898 | **0.8114** | 0.0000 | **** |
| **inferior_vena_cava** | 0.5370 | **0.5723** | 0.0000 | **** | 0.1976 | **0.2391** | 0.0000 | **** |
| **intervertebral_discs** | 0.4783 | **0.6174** | 0.0000 | **** | 0.3790 | **0.5059** | 0.0000 | **** |
| **kidney_left** | 0.8002 | **0.8224** | 0.0000 | **** | 0.6530 | **0.6746** | 0.0000 | **** |
| **kidney_right** | 0.8103 | **0.8291** | 0.0000 | **** | **0.7247** | 0.6057 | 0.0000 | **** |
| **liver** | 0.8818 | **0.8918** | 0.0000 | **** | **0.8511** | 0.8498 | 0.0010 | *** |
| **lung_left** | **0.9451** | 0.9086 | 0.0000 | **** | **0.9497** | 0.9362 | 0.0000 | **** |
| **lung_right** | **0.9571** | 0.9229 | 0.0000 | **** | **0.9614** | 0.9445 | 0.0000 | **** |
| **pancreas** | 0.3641 | **0.3653** | 0.2940 | ns | **0.0657** | 0.0588 | 0.0000 | **** |
| **portal_vein_and_splenic_vein** | 0.1796 | **0.2966** | 0.0000 | **** | NA | NA | NA | NA |
| **prostate** | 0.5696 | **0.6333** | 0.0000 | **** | **0.1449** | 0.0704 | 0.0000 | **** |
| **sacrum** | 0.7532 | **0.7583** | 0.0000 | **** | 0.6195 | **0.7094** | 0.0000 | **** |
| **scapula_left** | 0.5381 | **0.7053** | 0.0000 | **** | 0.4940 | **0.6530** | 0.0000 | **** |
| **scapula_right** | 0.4313 | **0.6134** | 0.0000 | **** | 0.4907 | **0.6597** | 0.0000 | **** |
| **small_bowel** | 0.3594 | **0.3959** | 0.0000 | **** | **0.2956** | 0.2944 | 1.0000 | ns |
| **spinal_cord** | 0.7645 | **0.7679** | 0.0022 | ** | 0.7223 | **0.7477** | 0.0000 | **** |
| **spleen** | 0.6992 | **0.6994** | 0.0000 | **** | 0.5381 | **0.6618** | 0.0000 | **** |
| **stomach** | **0.6257** | 0.5762 | 0.0000 | **** | 0.4089 | **0.4374** | 0.0000 | **** |
| **urinary_bladder** | **0.7718** | 0.6373 | 0.0000 | **** | **0.7901** | 0.6485 | 0.0000 | **** |
| **vertebrae** | 0.6757 | **0.7413** | 0.0000 | **** | 0.6196 | **0.6640** | 0.0000 | **** |

Supplementary Table J: Mean dice scores of the 71 masks segmented with VIBESegmentator for the female and male cohorts for the intensity-based and MIRTK registrations. The statistical tests were done using pairwise Wilcoxon signed-rank tests with adjustment for multiple testing using Bonferroni correction. (na: not applicable, ns: not significant, *: p-value < 0.05, **: p-value < 0.01, ***: p-value < 0.001, ****: p-value < 0.0001)

|  | **male** | | | | **female** | | | |
| --- | --- | --- | --- | --- | --- | --- | --- | --- |
| **mask name** | **intensity-based** | **MIRTK** | **p.adj** | **significance** | **intensity-based** | **MIRTK** | **p.adj** | **significance** |
| **adrenal gland left** | **0.4022** | 0.5632 | 0.000 | **** | **0.1718** | 0.3459 | 0.000 | **** |
| **adrenal gland right** | **0.3399** | 0.5567 | 0.000 | **** | **0.1639** | 0.3372 | 0.000 | **** |
| **aorta** | **0.7279** | 0.8467 | 0.000 | **** | 0.6393 | **0.7603** | 0.000 | **** |
| **atrial appendage left** | **0.4830** | 0.6055 | 0.000 | **** | **0.4388** | 0.3926 | 0.000 | **** |
| **autochthon left** | 0.8852 | **0.9179** | 0.000 | **** | 0.8349 | **0.9056** | 0.000 | **** |
| **autochthon right** | 0.8734 | **0.9097** | 0.000 | **** | 0.8260 | **0.9074** | 0.000 | **** |
| **bone other** | 0.5647 | **0.4908** | 0.000 | **** | 0.7167 | **0.6903** | 0.000 | **** |
| **brachiocephalic trunk** | 0.5509 | **0.6492** | 0.000 | **** | 0.5007 | **0.6156** | 0.000 | **** |
| **brachiocephalic vein left** | 0.5535 | **0.6300** | 0.000 | **** | 0.5058 | **0.6352** | 0.000 | **** |
| **brachiocephalic vein right** | 0.6335 | **0.6289** | 1.000 | ns | 0.5708 | **0.7224** | 0.000 | **** |
| **clavicula left** | 0.6674 | **0.6171** | 0.000 | **** | 0.6149 | **0.5898** | 0.020 | * |
| **clavicula right** | 0.6622 | **0.4781** | 0.000 | **** | 0.6508 | **0.6494** | 1.000 | ns |
| **common carotid artery left** | 0.5379 | **0.5517** | 0.001 | ** | 0.4609 | **0.4978** | 0.000 | **** |
| **common carotid artery right** | 0.4742 | **0.4363** | 0.000 | **** | 0.4577 | **0.5734** | 0.000 | **** |
| **costal cartilages** | 0.5471 | **0.5480** | 1.000 | ns | 0.3885 | **0.6387** | 0.000 | **** |
| **duodenum** | 0.4967 | **0.6427** | 0.000 | **** | 0.3766 | **0.4560** | 0.000 | **** |
| **esophagus** | 0.5826 | **0.7012** | 0.000 | **** | 0.5521 | **0.6724** | 0.000 | **** |
| **femur left** | 0.8724 | **0.8903** | 0.000 | **** | 0.8645 | **0.8932** | 0.000 | **** |
| **femur right** | 0.8675 | **0.8879** | 0.000 | **** | 0.8621 | **0.8885** | 0.000 | **** |
| **gallbladder** | 0.2044 | **0.3550** | 0.000 | **** | 0.1252 | **0.2107** | 0.000 | **** |
| **gluteus maximus left** | 0.8617 | **0.9297** | 0.000 | **** | 0.8657 | **0.9325** | 0.000 | **** |
| **gluteus maximus right** | 0.8881 | **0.9438** | 0.000 | **** | 0.8246 | **0.9332** | 0.000 | **** |
| **gluteus medius left** | 0.8559 | **0.8926** | 0.000 | **** | 0.8061 | **0.8827** | 0.000 | **** |
| **gluteus medius right** | 0.8510 | **0.8999** | 0.000 | **** | 0.7704 | **0.8786** | 0.000 | **** |
| **gluteus minimus left** | 0.8220 | **0.8488** | 0.000 | **** | 0.7573 | **0.8213** | 0.000 | **** |
| **gluteus minimus right** | 0.8075 | **0.8451** | 0.000 | **** | 0.7589 | **0.8235** | 0.000 | **** |
| **heart** | **0.8185** | 0.8876 | 0.000 | **** | **0.8296** | 0.8961 | 0.000 | **** |
| **hip left** | 0.8186 | **0.8625** | 0.000 | **** | 0.7588 | **0.8562** | 0.000 | **** |
| **hip right** | 0.8336 | **0.8818** | 0.000 | **** | 0.7258 | **0.8423** | 0.000 | **** |
| **humerus left** | 0.7869 | **0.8061** | 0.000 | **** | 0.7603 | **0.7408** | 0.000 | **** |
| **humerus right** | 0.7508 | **0.5791** | 0.000 | **** | 0.6993 | **0.7917** | 0.000 | **** |
| **iliac artery left** | 0.3436 | **0.5562** | 0.000 | **** | 0.4420 | **0.5802** | 0.000 | **** |
| **iliac artery right** | 0.3548 | **0.5087** | 0.000 | **** | 0.4501 | **0.5517** | 0.000 | **** |
| **iliac vena left** | 0.4519 | **0.6843** | 0.000 | **** | 0.4994 | **0.6840** | 0.000 | **** |
| **iliac vena right** | 0.4356 | **0.6117** | 0.000 | **** | 0.5411 | **0.6444** | 0.000 | **** |
| **iliopsoas left** | 0.8189 | **0.8848** | 0.000 | **** | 0.7518 | **0.8444** | 0.000 | **** |
| **iliopsoas right** | 0.8053 | **0.8808** | 0.000 | **** | 0.7399 | **0.8163** | 0.000 | **** |
| **inferior vena cava** | **0.6087** | 0.7368 | 0.000 | **** | 0.4614 | **0.5960** | 0.000 | **** |
| **inner fat** | 0.5350 | **0.7302** | 0.000 | **** | 0.4366 | **0.6303** | 0.000 | **** |
| **intestine** | 0.6201 | **0.7371** | 0.000 | **** | 0.7069 | **0.7489** | 0.000 | **** |
| **IVD** | 0.6210 | **0.6588** | 0.000 | **** | 0.4118 | **0.5405** | 0.000 | **** |
| **kidney left** | 0.6541 | **0.8531** | 0.000 | **** | **0.5605** | 0.7547 | 0.000 | **** |
| **kidney right** | 0.6221 | **0.8708** | 0.000 | **** | **0.5938** | 0.6965 | 0.000 | **** |
| **liver** | 0.8242 | **0.9103** | 0.000 | **** | 0.7980 | **0.8677** | 0.000 | **** |
| **lung lower lobe left** | 0.7725 | **0.8317** | 0.000 | **** | 0.8056 | **0.8960** | 0.000 | **** |
| **lung lower lobe right** | **0.8127** | 0.8466 | 0.000 | **** | 0.8528 | **0.8796** | 0.000 | **** |
| **lung middle lobe right** | 0.7459 | **0.8250** | 0.000 | **** | 0.7381 | **0.8284** | 0.000 | **** |
| **lung upper lobe left** | 0.8706 | **0.8963** | 0.000 | **** | 0.8563 | **0.9038** | 0.000 | **** |
| **lung upper lobe right** | 0.8487 | **0.8813** | 0.000 | **** | 0.8538 | **0.9022** | 0.000 | **** |
| **muscle** | 0.8231 | **0.8867** | 0.000 | **** | 0.7978 | **0.8769** | 0.000 | **** |
| **pancreas** | 0.5243 | **0.6206** | 0.000 | **** | **0.3208** | 0.3445 | 0.000 | *** |
| **portal vein and splenic vein** | 0.3939 | **0.5514** | 0.000 | **** | **0.2833** | 0.2840 | 1.000 | ns |
| **prostate** | 0.6175 | **0.6197** | 1.000 | ns | NA | NA | NA | NA |
| **pulmonary vein** | **0.5851** | 0.6227 | 0.000 | **** | 0.5908 | 0.6088 | 0.000 | **** |
| **sacrum** | 0.8403 | **0.8850** | 0.000 | **** | **0.8040** | 0.8374 | 0.000 | **** |
| **scapula left** | 0.7722 | **0.7844** | 0.000 | **** | 0.7003 | **0.6673** | 0.000 | **** |
| **scapula right** | 0.7284 | **0.6238** | 0.000 | **** | 0.6921 | **0.7050** | 0.000 | **** |
| **spinal channel** | 0.6909 | **0.7337** | 0.000 | **** | 0.4940 | **0.7138** | 0.000 | **** |
| **spinal cord** | 0.5622 | **0.6449** | 0.000 | **** | 0.4678 | **0.6507** | 0.000 | **** |
| **spleen** | **0.4957** | 0.7001 | 0.000 | **** | 0.5162 | **0.7005** | 0.000 | **** |
| **sternum** | 0.7157 | **0.6111** | 0.000 | **** | **0.6461** | 0.7636 | 0.000 | **** |
| **stomach** | **0.5026** | 0.6393 | 0.000 | **** | 0.4130 | **0.4958** | 0.000 | **** |
| **subclavian artery left** | 0.5667 | **0.5770** | 0.497 | ns | 0.5318 | **0.5259** | 1.000 | ns |
| **subclavian artery right** | 0.5296 | **0.5567** | 0.000 | **** | 0.5403 | **0.6519** | 0.000 | **** |
| **subcutaneous fat** | 0.7751 | **0.8795** | 0.000 | **** | 0.8240 | **0.9169** | 0.000 | **** |
| **superior vena cava** | 0.6578 | **0.7209** | 0.000 | **** | 0.6016 | **0.7825** | 0.000 | **** |
| **thyroid gland** | 0.5087 | **0.4876** | 1.000 | ns | **0.4455** | 0.5558 | 0.000 | **** |
| **trachea** | 0.6730 | **0.7283** | 0.000 | **** | 0.5838 | **0.7058** | 0.000 | **** |
| **urinary bladder** | 0.5699 | **0.6281** | 0.000 | **** | 0.6191 | **0.6975** | 0.000 | **** |
| **vertebra body** | 0.7601 | **0.7925** | 0.000 | **** | 0.6032 | **0.7165** | 0.000 | **** |
| **vertebra posterior elements** | 0.6488 | **0.6254** | 0.000 | **** | 0.4961 | **0.6145** | 0.000 | **** |

Supplementary Table K: Mean dice scores of the 71 masks segmented with VIBESegmentator for the female and male cohorts for the mask-supported and MIRTK registrations. The statistical tests were done using pairwise Wilcoxon signed-rank tests with adjustment for multiple testing using Bonferroni correction. (na: not applicable, ns: not significant, *: p-value < 0.05, **: p-value < 0.01, ***: p-value < 0.001, ****: p-value < 0.0001)

|  | **male** | | | | **female** | | | |
| --- | --- | --- | --- | --- | --- | --- | --- | --- |
| **mask name** | **MIRTK** | **mask-supported** | **p.adj** | **significance** | **MIRTK** | **mask-supported** | **p.adj** | **significance** |
| **adrenal gland left** | 0.4022 | **0.5631** | 0.000 | **** | 0.1718 | **0.3380** | 0.000 | **** |
| **adrenal gland right** | 0.3399 | **0.5573** | 0.000 | **** | 0.1639 | **0.3292** | 0.000 | **** |
| **aorta** | 0.7279 | **0.8466** | 0.000 | **** | 0.6393 | **0.7649** | 0.000 | **** |
| **atrial appendage left** | 0.4830 | **0.5988** | 0.000 | **** | **0.4388** | 0.3924 | 0.000 | **** |
| **autochthon left** | 0.8852 | **0.9514** | 0.000 | **** | 0.8349 | **0.9397** | 0.000 | **** |
| **autochthon right** | 0.8734 | **0.9526** | 0.000 | **** | 0.8260 | **0.9468** | 0.000 | **** |
| **bone other** | 0.5647 | **0.6070** | 0.000 | **** | 0.7167 | **0.8310** | 0.000 | **** |
| **brachiocephalic trunk** | 0.5509 | **0.6817** | 0.000 | **** | 0.5007 | **0.6278** | 0.000 | **** |
| **brachiocephalic vein left** | 0.5535 | **0.6774** | 0.000 | **** | 0.5058 | **0.6653** | 0.000 | **** |
| **brachiocephalic vein right** | 0.6335 | **0.7202** | 0.000 | **** | 0.5708 | **0.7340** | 0.000 | **** |
| **clavicula left** | 0.6674 | **0.8372** | 0.000 | **** | 0.6149 | **0.8031** | 0.000 | **** |
| **clavicula right** | 0.6622 | **0.8131** | 0.000 | **** | 0.6508 | **0.8541** | 0.000 | **** |
| **common carotid artery left** | 0.5379 | **0.7214** | 0.000 | **** | 0.4609 | **0.6460** | 0.000 | **** |
| **common carotid artery right** | 0.4742 | **0.7618** | 0.000 | **** | 0.4577 | **0.7743** | 0.000 | **** |
| **costal cartilages** | 0.5471 | **0.7067** | 0.000 | **** | 0.3885 | **0.7535** | 0.000 | **** |
| **duodenum** | 0.4967 | **0.6610** | 0.000 | **** | 0.3766 | **0.4641** | 0.000 | **** |
| **esophagus** | 0.5826 | **0.7396** | 0.000 | **** | 0.5521 | **0.6969** | 0.000 | **** |
| **femur left** | 0.8724 | **0.9281** | 0.000 | **** | 0.8645 | **0.9656** | 0.000 | **** |
| **femur right** | 0.8675 | **0.9042** | 0.000 | **** | 0.8621 | **0.9602** | 0.000 | **** |
| **gallbladder** | 0.2044 | **0.3624** | 0.000 | **** | 0.1252 | **0.2199** | 0.000 | **** |
| **gluteus maximus left** | 0.8617 | **0.9651** | 0.000 | **** | 0.8657 | **0.9704** | 0.000 | **** |
| **gluteus maximus right** | 0.8881 | **0.9662** | 0.000 | **** | 0.8246 | **0.9717** | 0.000 | **** |
| **gluteus medius left** | 0.8559 | **0.9197** | 0.000 | **** | 0.8061 | **0.9155** | 0.000 | **** |
| **gluteus medius right** | 0.8510 | **0.9184** | 0.000 | **** | 0.7704 | **0.9124** | 0.000 | **** |
| **gluteus minimus left** | 0.8220 | **0.8678** | 0.000 | **** | 0.7573 | **0.8512** | 0.000 | **** |
| **gluteus minimus right** | 0.8075 | **0.8589** | 0.000 | **** | 0.7589 | **0.8569** | 0.000 | **** |
| **heart** | 0.8185 | **0.8876** | 0.000 | **** | 0.8296 | **0.8947** | 0.000 | **** |
| **hip left** | 0.8186 | **0.8991** | 0.000 | **** | 0.7588 | **0.9053** | 0.000 | **** |
| **hip right** | 0.8336 | **0.9015** | 0.000 | **** | 0.7258 | **0.8975** | 0.000 | **** |
| **humerus left** | 0.7869 | **0.9283** | 0.000 | **** | 0.7603 | **0.9209** | 0.000 | **** |
| **humerus right** | 0.7508 | **0.8026** | 0.000 | **** | 0.6993 | **0.9265** | 0.000 | **** |
| **iliac artery left** | 0.3436 | **0.5825** | 0.000 | **** | 0.4420 | **0.5986** | 0.000 | **** |
| **iliac artery right** | 0.3548 | **0.5375** | 0.000 | **** | 0.4501 | **0.5890** | 0.000 | **** |
| **iliac vena left** | 0.4519 | **0.6999** | 0.000 | **** | 0.4994 | **0.7021** | 0.000 | **** |
| **iliac vena right** | 0.4356 | **0.6239** | 0.000 | **** | 0.5411 | **0.6669** | 0.000 | **** |
| **iliopsoas left** | 0.8189 | **0.8999** | 0.000 | **** | 0.7518 | **0.8637** | 0.000 | **** |
| **iliopsoas right** | 0.8053 | **0.8916** | 0.000 | **** | 0.7399 | **0.8403** | 0.000 | **** |
| **inferior vena cava** | 0.6087 | **0.7339** | 0.000 | **** | 0.4614 | **0.5991** | 0.000 | **** |
| **inner fat** | 0.5350 | **0.7684** | 0.000 | **** | 0.4366 | **0.6910** | 0.000 | **** |
| **intestine** | 0.6201 | **0.7818** | 0.000 | **** | 0.7069 | **0.7963** | 0.000 | **** |
| **IVD** | 0.6210 | **0.6615** | 0.000 | **** | 0.4118 | **0.5463** | 0.000 | **** |
| **kidney left** | 0.6541 | **0.8636** | 0.000 | **** | 0.5605 | **0.7452** | 0.000 | **** |
| **kidney right** | 0.6221 | **0.8753** | 0.000 | **** | 0.5938 | **0.6969** | 0.000 | **** |
| **liver** | 0.8242 | **0.9143** | 0.000 | **** | 0.7980 | **0.8722** | 0.000 | **** |
| **lung lower lobe left** | 0.7725 | **0.8343** | 0.000 | **** | 0.8056 | **0.9018** | 0.000 | **** |
| **lung lower lobe right** | 0.8127 | **0.8468** | 0.000 | **** | 0.8528 | **0.8858** | 0.000 | **** |
| **lung middle lobe right** | 0.7459 | **0.8313** | 0.000 | **** | 0.7381 | **0.8369** | 0.000 | **** |
| **lung upper lobe left** | 0.8706 | **0.9092** | 0.000 | **** | 0.8563 | **0.9171** | 0.000 | **** |
| **lung upper lobe right** | 0.8487 | **0.8924** | 0.000 | **** | 0.8538 | **0.9121** | 0.000 | **** |
| **muscle** | 0.8231 | **0.9647** | 0.000 | **** | 0.7978 | **0.9658** | 0.000 | **** |
| **pancreas** | 0.5243 | **0.6298** | 0.000 | **** | 0.3208 | **0.3483** | 0.000 | **** |
| **portal vein and splenic vein** | 0.3939 | **0.5564** | 0.000 | **** | 0.2833 | 0.2775 | 1.000 | ns |
| **prostate** | 0.6175 | **0.6759** | 0.000 | **** | NA | NA | NA | NA |
| **pulmonary vein** | 0.5851 | **0.6217** | 0.000 | **** | 0.5908 | **0.6068** | 0.000 | **** |
| **sacrum** | 0.8403 | **0.8963** | 0.000 | **** | 0.8040 | **0.8592** | 0.000 | **** |
| **scapula left** | 0.7722 | **0.9411** | 0.000 | **** | 0.7003 | **0.8947** | 0.000 | **** |
| **scapula right** | 0.7284 | **0.8781** | 0.000 | **** | 0.6921 | **0.9077** | 0.000 | **** |
| **spinal channel** | 0.6909 | **0.7500** | 0.000 | **** | 0.4940 | **0.7259** | 0.000 | **** |
| **spinal cord** | 0.5622 | **0.6762** | 0.000 | **** | 0.4678 | **0.6697** | 0.000 | **** |
| **spleen** | 0.4957 | **0.7064** | 0.000 | **** | 0.5162 | **0.6985** | 0.000 | **** |
| **sternum** | 0.7157 | 0.7286 | 1.000 | ns | 0.6461 | **0.8474** | 0.000 | **** |
| **stomach** | 0.5026 | **0.6361** | 0.000 | **** | 0.4130 | **0.5070** | 0.000 | **** |
| **subclavian artery left** | 0.5667 | **0.7504** | 0.000 | **** | 0.5318 | **0.6708** | 0.000 | **** |
| **subclavian artery right** | 0.5296 | **0.7990** | 0.000 | **** | 0.5403 | **0.7993** | 0.000 | **** |
| **subcutaneous fat** | 0.7751 | **0.9628** | 0.000 | **** | 0.8240 | **0.9756** | 0.000 | **** |
| **superior vena cava** | 0.6578 | **0.7281** | 0.000 | **** | 0.6016 | **0.7826** | 0.000 | **** |
| **thyroid gland** | 0.5087 | **0.8294** | 0.000 | **** | 0.4455 | **0.8086** | 0.000 | **** |
| **trachea** | 0.6730 | **0.7985** | 0.000 | **** | 0.5838 | **0.7455** | 0.000 | **** |
| **urinary bladder** | 0.5699 | **0.6433** | 0.000 | **** | 0.6191 | **0.7086** | 0.000 | **** |
| **vertebra body** | 0.7601 | **0.8001** | 0.000 | **** | 0.6032 | **0.7252** | 0.000 | **** |
| **vertebra posterior elements** | 0.6488 | 0.6495 | 1.000 | ns | 0.4961 | **0.6329** | 0.000 | **** |

Supplementary Table L: Mean dice scores of the 40 masks segmented with MRSegmentator for the female and male cohorts for the mask-supported and MIRTK registrations. The statistical tests were done using pairwise Wilcoxon signed-rank tests with adjustment for multiple testing using Bonferroni correction. (na: not applicable, ns: not significant, *: p-value < 0.05, **: p-value < 0.01, ***: p-value < 0.001, ****: p-value < 0.0001)

|  | **male** | | | | **female** | | | |
| --- | --- | --- | --- | --- | --- | --- | --- | --- |
| **mask name** | **MIRTK** | **mask-supported** | **p.adj** | **significance** | **MIRTK** | **mask-supported** | **p.adj** | **significance** |
| **aorta** | 0.6884 | **0.8476** | 0.0000 | **** | 0.6693 | **0.7701** | 0.0000 | **** |
| **colon** | 0.3158 | **0.5122** | 0.0000 | **** | **0.4404** | 0.4312 | 0.0000 | **** |
| **duodenum** | 0.3712 | **0.5616** | 0.0000 | **** | 0.3257 | **0.4316** | 0.0000 | **** |
| **esophagus** | 0.4342 | **0.6550** | 0.0000 | **** | 0.5194 | **0.5689** | 0.0000 | **** |
| **gallbladder** | 0.1513 | **0.2485** | 0.0000 | **** | **0.1671** | 0.1198 | 0.0000 | **** |
| **heart** | 0.7938 | **0.8880** | 0.0000 | **** | 0.8424 | **0.8835** | 0.0000 | **** |
| **inferior_vena_cava** | 0.5072 | **0.6760** | 0.0000 | **** | 0.3798 | **0.4965** | 0.0000 | **** |
| **left_adrenal_gland** | 0.3042 | **0.5170** | 0.0000 | **** | NA | NA | NA | NA |
| **left_autochthonous_muscle** | 0.8754 | **0.9102** | 0.0000 | **** | 0.8944 | **0.9017** | 0.0000 | **** |
| **left_femur** | 0.8541 | **0.8838** | 0.0000 | **** | 0.8775 | **0.9151** | 0.0000 | **** |
| **left_gluteus_maximus** | 0.8396 | **0.9234** | 0.0000 | **** | 0.9162 | **0.9247** | 0.0000 | **** |
| **left_gluteus_medius** | 0.8528 | **0.8878** | 0.0000 | **** | 0.8562 | **0.8910** | 0.0000 | **** |
| **left_gluteus_minimus** | 0.7790 | **0.8054** | 0.0000 | **** | 0.7315 | **0.7720** | 0.0000 | **** |
| **left_hip** | 0.8037 | **0.8676** | 0.0000 | **** | 0.7862 | **0.8706** | 0.0000 | **** |
| **left_iliac_artery** | 0.2369 | **0.4966** | 0.0000 | **** | 0.3305 | **0.4952** | 0.0000 | **** |
| **left_iliac_vena** | 0.3771 | **0.6530** | 0.0000 | **** | 0.5197 | **0.6830** | 0.0000 | **** |
| **left_iliopsoas_muscle** | 0.8112 | **0.8873** | 0.0000 | **** | 0.8144 | **0.8490** | 0.0000 | **** |
| **left_kidney** | 0.6588 | **0.8672** | 0.0000 | **** | 0.7224 | **0.7473** | 0.0000 | **** |
| **left_lung** | 0.8603 | **0.9097** | 0.0000 | **** | **0.9512** | 0.9390 | 0.0000 | **** |
| **liver** | 0.8292 | **0.9091** | 0.0000 | **** | 0.8533 | **0.8673** | 0.0000 | **** |
| **pancreas** | 0.4045 | **0.5279** | 0.0000 | **** | 0.2786 | **0.3001** | 0.0000 | **** |
| **portal_vein_and_splenic_vein** | 0.2334 | **0.3713** | 0.0000 | **** | **0.1907** | 0.1479 | 0.0000 | **** |
| **right_adrenal_gland** | 0.2794 | **0.4989** | 0.0000 | **** | 0.1366 | **0.2147** | 0.0000 | **** |
| **right_autochthonous_muscle** | 0.8599 | **0.8982** | 0.0000 | **** | **0.8927** | 0.8869 | 0.0000 | **** |
| **right_femur** | 0.8538 | **0.8798** | 0.0000 | **** | 0.8727 | **0.9110** | 0.0000 | **** |
| **right_gluteus_maximus** | 0.8802 | **0.9360** | 0.0000 | **** | 0.9084 | **0.9260** | 0.0000 | **** |
| **right_gluteus_medius** | 0.8664 | **0.9046** | 0.0000 | **** | 0.8523 | **0.8895** | 0.0000 | **** |
| **right_gluteus_minimus** | 0.7952 | **0.8237** | 0.0000 | **** | 0.7661 | **0.7881** | 0.0000 | **** |
| **right_hip** | 0.8112 | **0.8763** | 0.0000 | **** | 0.7684 | **0.8613** | 0.0000 | **** |
| **right_iliac_artery** | 0.2038 | **0.4013** | 0.0000 | **** | 0.2639 | **0.4856** | 0.0000 | **** |
| **right_iliac_vena** | 0.3269 | **0.5453** | 0.0000 | **** | 0.3789 | **0.5931** | 0.0000 | **** |
| **right_iliopsoas_muscle** | 0.7836 | **0.8819** | 0.0000 | **** | 0.8061 | **0.8372** | 0.0000 | **** |
| **right_kidney** | 0.6215 | **0.8824** | 0.0000 | **** | **0.7831** | 0.6877 | 0.0000 | **** |
| **right_lung** | 0.8699 | **0.9223** | 0.0000 | **** | **0.9624** | 0.9406 | 0.0000 | **** |
| **sacrum** | 0.7696 | **0.8482** | 0.0000 | **** | 0.7061 | **0.8101** | 0.0000 | **** |
| **small_bowel** | 0.3919 | **0.5639** | 0.0000 | **** | 0.5015 | **0.5325** | 0.0000 | **** |
| **spine** | 0.7528 | **0.7831** | 0.0000 | **** | 0.6142 | **0.6946** | 0.0000 | **** |
| **spleen** | 0.5041 | **0.7186** | 0.0000 | **** | 0.5924 | **0.7002** | 0.0000 | **** |
| **stomach** | 0.4603 | **0.5982** | 0.0000 | **** | 0.4224 | **0.4616** | 0.0000 | **** |
| **urinary_bladder** | 0.5799 | **0.6291** | 0.0000 | **** | **0.8065** | 0.6687 | 0.0000 | **** |

Supplementary Table M: Mean dice scores of the 50 masks segmented with TotalSegmentator for the female and male cohorts for the mask-supported and MIRTK registrations. The statistical tests were done using pairwise Wilcoxon signed-rank tests with adjustment for multiple testing using Bonferroni correction. (na: not applicable, ns: not significant, *: p-value < 0.05, **: p-value < 0.01, ***: p-value < 0.001, ****: p-value < 0.0001)

|  | **male** | | | | **female** | | | |
| --- | --- | --- | --- | --- | --- | --- | --- | --- |
| **mask name** | **MIRTK** | **mask-supported** | **p.adj** | **significance** | **MIRTK** | **mask-supported** | **p.adj** | **significance** |
| **adrenal_gland_left** | 0.2696 | **0.4339** | 0.0000 | **** | 0.0623 | **0.1384** | 0.0000 | **** |
| **adrenal_gland_right** | 0.2518 | **0.4516** | 0.0000 | **** | 0.0853 | **0.2034** | 0.0000 | **** |
| **aorta** | 0.6183 | **0.8005** | 0.0000 | **** | 0.5296 | **0.7062** | 0.0000 | **** |
| **autochthon_left** | 0.8710 | **0.9068** | 0.0000 | **** | 0.8146 | **0.8929** | 0.0000 | **** |
| **autochthon_right** | 0.8526 | **0.8994** | 0.0000 | **** | 0.7914 | **0.8762** | 0.0000 | **** |
| **clavicula_left** | 0.4271 | **0.5717** | 0.0000 | **** | 0.3589 | **0.5150** | 0.0000 | **** |
| **clavicula_right** | 0.3905 | **0.5050** | 0.0000 | **** | 0.3447 | **0.5156** | 0.0000 | **** |
| **colon** | 0.2882 | **0.4759** | 0.0000 | **** | 0.2619 | **0.3747** | 0.0000 | **** |
| **duodenum** | 0.2870 | **0.4763** | 0.0000 | **** | 0.1481 | **0.2574** | 0.0000 | **** |
| **esophagus** | 0.4220 | **0.6156** | 0.0000 | **** | 0.3999 | **0.5442** | 0.0000 | **** |
| **femur_left** | 0.8380 | **0.8722** | 0.0000 | **** | 0.8256 | **0.8972** | 0.0000 | **** |
| **femur_right** | 0.8414 | **0.8758** | 0.0000 | **** | 0.8370 | **0.9038** | 0.0000 | **** |
| **gallbladder** | 0.1395 | **0.2269** | 0.0000 | **** | 0.0611 | **0.1199** | 0.0000 | **** |
| **gluteus_maximus_left** | 0.8415 | **0.9145** | 0.0000 | **** | 0.8510 | **0.9189** | 0.0000 | **** |
| **gluteus_maximus_right** | 0.8686 | **0.9178** | 0.0000 | **** | 0.7817 | **0.9069** | 0.0000 | **** |
| **gluteus_medius_left** | 0.7958 | **0.8269** | 0.0000 | **** | 0.6907 | **0.7589** | 0.0000 | **** |
| **gluteus_medius_right** | 0.8077 | **0.8455** | 0.0000 | **** | 0.6390 | **0.7458** | 0.0000 | **** |
| **gluteus_minimus_left** | 0.7391 | **0.7703** | 0.0000 | **** | 0.6550 | **0.7179** | 0.0000 | **** |
| **gluteus_minimus_right** | 0.7352 | **0.7644** | 0.0000 | **** | 0.6436 | **0.7219** | 0.0000 | **** |
| **heart** | 0.7826 | **0.8678** | 0.0000 | **** | 0.7900 | **0.8632** | 0.0000 | **** |
| **hip_left** | 0.7274 | **0.8251** | 0.0000 | **** | 0.6486 | **0.8027** | 0.0000 | **** |
| **hip_right** | 0.7362 | **0.8312** | 0.0000 | **** | 0.5901 | **0.7971** | 0.0000 | **** |
| **humerus_left** | 0.7410 | **0.8263** | 0.0000 | **** | 0.6942 | **0.8010** | 0.0000 | **** |
| **humerus_right** | **0.7116** | 0.6805 | 0.0000 | **** | 0.6359 | **0.8181** | 0.0000 | **** |
| **iliac_artery_left** | 0.2005 | **0.4032** | 0.0000 | **** | 0.1809 | **0.3290** | 0.0000 | **** |
| **iliac_artery_right** | 0.1702 | **0.3359** | 0.0000 | **** | 0.1805 | **0.3546** | 0.0000 | **** |
| **iliac_vena_left** | 0.3268 | **0.5562** | 0.0000 | **** | 0.3542 | **0.5605** | 0.0000 | **** |
| **iliac_vena_right** | 0.2882 | **0.5024** | 0.0000 | **** | 0.3446 | **0.4995** | 0.0000 | **** |
| **iliopsoas_left** | 0.7681 | **0.8563** | 0.0000 | **** | 0.6931 | **0.8243** | 0.0000 | **** |
| **iliopsoas_right** | 0.7523 | **0.8666** | 0.0000 | **** | 0.6756 | **0.8114** | 0.0000 | **** |
| **inferior_vena_cava** | 0.4507 | **0.5723** | 0.0000 | **** | 0.1336 | **0.2391** | 0.0000 | **** |
| **intervertebral_discs** | 0.5953 | **0.6174** | 0.0000 | **** | 0.3902 | **0.5059** | 0.0000 | **** |
| **kidney_left** | 0.5677 | **0.8224** | 0.0000 | **** | 0.4288 | **0.6746** | 0.0000 | **** |
| **kidney_right** | 0.5443 | **0.8291** | 0.0000 | **** | 0.4806 | **0.6057** | 0.0000 | **** |
| **liver** | 0.8101 | **0.8918** | 0.0000 | **** | 0.7915 | **0.8498** | 0.0000 | **** |
| **lung_left** | 0.8538 | **0.9086** | 0.0000 | **** | 0.8590 | **0.9362** | 0.0000 | **** |
| **lung_right** | 0.8643 | **0.9229** | 0.0000 | **** | 0.8866 | **0.9445** | 0.0000 | **** |
| **pancreas** | 0.2529 | **0.3653** | 0.0000 | **** | 0.0476 | **0.0588** | 0.0000 | **** |
| **portal_vein_and_splenic_vein** | 0.1735 | **0.2966** | 0.0000 | **** | **0.0783** | 0.0704 | 0.0480 | * |
| **prostate** | 0.5628 | **0.6333** | 0.0000 | **** | NA | NA | NA | NA |
| **sacrum** | 0.6799 | **0.7583** | 0.0000 | **** | 0.6370 | **0.7094** | 0.0000 | **** |
| **scapula_left** | 0.5832 | **0.7053** | 0.0000 | **** | 0.5117 | **0.6530** | 0.0000 | **** |
| **scapula_right** | 0.5384 | **0.6134** | 0.0000 | **** | 0.5213 | **0.6597** | 0.0000 | **** |
| **small_bowel** | 0.2511 | **0.3959** | 0.0000 | **** | 0.2344 | **0.2944** | 0.0000 | **** |
| **spinal_cord** | 0.7145 | **0.7679** | 0.0000 | **** | 0.4775 | **0.7477** | 0.0000 | **** |
| **spleen** | 0.4866 | **0.6994** | 0.0000 | **** | 0.4746 | **0.6618** | 0.0000 | **** |
| **stomach** | 0.4375 | **0.5762** | 0.0000 | **** | 0.3404 | **0.4374** | 0.0000 | **** |
| **urinary_bladder** | 0.5808 | **0.6373** | 0.0000 | **** | 0.5824 | **0.6485** | 0.0000 | **** |
| **vertebrae** | 0.7143 | **0.7413** | 0.0000 | **** | 0.5444 | **0.6640** | 0.0000 | **** |
